# m6A RNA methylation of major satellite repeat transcripts facilitates chromatin association and RNA:DNA hybrid formation in mouse heterochromatin

**DOI:** 10.1093/nar/gkab364

**Published:** 2021-05-17

**Authors:** Katarzyna J Duda, Reagan W Ching, Lisa Jerabek, Nicholas Shukeir, Galina Erikson, Bettina Engist, Megumi Onishi-Seebacher, Valentina Perrera, Florian Richter, Gerhard Mittler, Katharina Fritz, Mark Helm, Philip Knuckles, Marc Bühler, Thomas Jenuwein

**Affiliations:** Max Planck Institute of Immunobiology and Epigenetics, Freiburg 79108, Germany; Max Planck Institute of Immunobiology and Epigenetics, Freiburg 79108, Germany; Max Planck Institute of Immunobiology and Epigenetics, Freiburg 79108, Germany; Max Planck Institute of Immunobiology and Epigenetics, Freiburg 79108, Germany; Max Planck Institute of Immunobiology and Epigenetics, Freiburg 79108, Germany; Max Planck Institute of Immunobiology and Epigenetics, Freiburg 79108, Germany; Max Planck Institute of Immunobiology and Epigenetics, Freiburg 79108, Germany; Max Planck Institute of Immunobiology and Epigenetics, Freiburg 79108, Germany; Max Planck Institute of Immunobiology and Epigenetics, Freiburg 79108, Germany; Institute of Pharmaceutical and Biomedical Sciences, Johannes Gutenberg – University, Mainz 55128, Germany; Max Planck Institute of Immunobiology and Epigenetics, Freiburg 79108, Germany; Institute of Pharmaceutical and Biomedical Sciences, Johannes Gutenberg – University, Mainz 55128, Germany; Institute of Pharmaceutical and Biomedical Sciences, Johannes Gutenberg – University, Mainz 55128, Germany; Friedrich Miescher Institute for Biomedical Research, Basel, 4058, Switzerland and University of Basel, Basel 4051, Switzerland; Friedrich Miescher Institute for Biomedical Research, Basel, 4058, Switzerland and University of Basel, Basel 4051, Switzerland; Max Planck Institute of Immunobiology and Epigenetics, Freiburg 79108, Germany

## Abstract

Heterochromatin has essential functions in maintaining chromosome structure, in protecting genome integrity and in stabilizing gene expression programs. Heterochromatin is often nucleated by underlying DNA repeat sequences, such as major satellite repeats (MSR) and long interspersed nuclear elements (LINE). In order to establish heterochromatin, MSR and LINE elements need to be transcriptionally competent and generate non-coding repeat RNA that remain chromatin associated. We explored whether these heterochromatic RNA, similar to DNA and histones, may be methylated, particularly for 5-methylcytosine (5mC) or methyl-6-adenosine (m6A). Our analysis in mouse ES cells identifies only background level of 5mC but significant enrichment for m6A on heterochromatic RNA. Moreover, MSR transcripts are a novel target for m6A RNA modification, and their m6A RNA enrichment is decreased in ES cells that are mutant for *Mettl3* or *Mettl14*, which encode components of a central RNA methyltransferase complex. Importantly, MSR transcripts that are partially deficient in m6A RNA methylation display impaired chromatin association and have a reduced potential to form RNA:DNA hybrids. We propose that m6A modification of MSR RNA will enhance the functions of MSR repeat transcripts to stabilize mouse heterochromatin.

## INTRODUCTION

Constitutive heterochromatin spans the pericentric region of each mouse chromosome. It is defined by underlying repeat DNA sequences, such as the major satellite repeats (MSR) and is characterized by DNA methylation, H3K9me3 methylation and heterochromatin protein 1 (HP1) accumulation (1,2). The basic unit of an MSR DNA repeat is 234 bp long, AT-rich and consists of four sub-repeats (3,4). MSR units are organized in arrays of > 10,000 re-iterated copies in the pericentric regions of each mouse chromosome, accounting for ∼3.6% of the DNA sequence in the mouse genome (5). A minor fraction of the MSR repeats remain transcriptionally competent and are bi-directionally transcribed by RNA polymerase II (6,7). MSR transcripts are chromatin-associated and form RNA:DNA hybrids, which facilitate the retention of HP1 proteins (8,9) and Suv39h enzymes (7,10,11), demonstrating that MSR repeat RNA is a structural component of mouse heterochromatin.

Suv39h-dependent H3K9me3 also occurs over intact 5′ untranslated regions (5′UTR) of long interspersed nuclear elements (LINE), in particular the L1MdA subfamily (12). The 5′UTR of L1MdA contains a 208 bp unit that can be re-iterated up to 43 times. LINE elements can also be transcribed from either the sense or antisense strand and a subpopulation of truncated (i.e. lacking open reading frames) LINE transcripts remains chromatin-associated (7,12,13).

MSR and LINE transcription are important after fertilization to ensure development of the early mouse embryo (14–16), and MSR transcripts are required to initiate heterochromatin formation (14,15,17). In human cancer, satellite repeat transcripts were found to be upregulated in several tumors (18,19) where they can cause repeat expansions at pericentric heterochromatin via aberrant RNA:DNA hybrid formation (20). Together, these implications underscore both the physiological and pathological functions of satellite repeat RNA. However, our understanding of the transcriptional regulation of MSR repeat DNA and of the physico-chemical properties of MSR repeat transcripts is still limited.

RNA:DNA hybrids are often found as a duplex of nucleic acids, where RNA associates with complementary single stranded DNA while the second DNA strand is displaced, forming a structure called R-loop (21,22). R-loops have been implicated in transcriptional initiation and termination, thus regulating gene expression (23–25). When R-loops are not resolved properly, their accumulation leads to DNA damage and/or replication fork stalling and causes genomic instabilities (21,22). A study in *Caenorhabditis elegans* has shown that R-loops are suppressed by H3K9 methylation, which protects the genome from inappropriate repeat element expression and aberrant increase in RNA:DNA hybrid formation (26).

We became interested to explore whether heterochromatic RNA (hetRNA), in particular MSR and LINE L1MdA 5′UTR transcripts, are methylated, similar to DNA and histone methylation. 5-methylcytidine (5mC) was shown to be mostly present on tRNA and rRNA (27–29) and to some extent on non-coding RNA (30,31) and mRNA (32,33). N6-methyladenosine (m6A) is best studied on poly(A) transcripts, where it is mostly found at the 3′UTR (34,35). m6A is deposited by the Mettl3/Mettl14 (methyltransferase-like 3/14) complex, where Mettl3 is the catalytically active enzyme, while Mettl14 is directing substrate binding and complex stability (36–38). The importance of m6A modification of RNA has been well documented in regulating mRNA homeostasis (39,40) and translational activation (41–44). In addition, m6A RNA has been involved in modulating cell fate transitions and stem cell renewal (45–47), embryonic development (48,49), *Xist*-mediated X chromosome inactivation (50), and silencing of IAP retrotransposons (51,52).

In this study, we identified m6A as an abundant RNA modification of MSR and LINE L1MdA 5′UTR transcripts. We established that the Mettl3/Mettl14 complex deposits m6A methylation on hetRNA *in vitro* and is also relevant to direct m6A RNA methylation to hetRNA in mouse ES cells. Using *Mettl14* and *Metll3* null ES cell lines, we found that the decrease of m6A RNA methylation leads to impaired chromatin association of MSR transcripts and reduces their ability to form RNA:DNA hybrids. Our work identifies MSR repeat RNA as a novel target for m6A RNA modification and suggests that m6A methylation of MSR hetRNA strengthens the functions of MSR repeat transcripts in stabilizing heterochromatin integrity.

## MATERIALS AND METHODS

### Cell culture of mouse ES cells

Mouse embryonic stem (ES) cells were cultured on dishes coated with 2% gelatin in high glucose DMEM medium (Sigma-Aldrich) containing 15% Serum Replacement (Thermo Fisher Scientific), 100 U/ml penicillin, 100 μg/μl streptomycin (Sigma-Aldrich), 2 mM L-glutamine (Sigma-Aldrich), 0.1 mM beta-mercaptoethanol, 1× non-essential amino acids (Sigma-Aldrich) and 1 mM Na-pyruvate (Sigma-Aldrich). 1 ml of conditioned medium from COS-7 cells (ATCC, cat # CRl-1651) expressing Leukemia Inhibitory Factor (LIF) was added to 500 ml of ES cell culture medium to prevent ES cell differentiation. Cells were cultured at 37°C in 5% CO_2_.

### Isolation and purification of nuclear RNA

Nuclear RNA preparation was done according to published protocols (53). Nuclei from 2 × 10^7^ ES cells were lysed in 1 ml TRIreagent (Sigma-Aldrich) with 5–10 strokes of a syringe using 20, 23 and 26 G needles. RNA was precipitated with isopropanol, washed with 75% ethanol, resuspended in nuclease-free water (Qiagen) and stored at –80°C. The quality of the RNA was controlled using a Bioanalyzer RNA 6000 Nano Kit (Agilent). 20 μg of RNA was digested for 1 h at 37°C with 7 U of TURBO DNase I (Thermo Fisher Scientific), RNA was purified using the RNeasy MinElute Cleanup kit (Qiagen) and the eluted RNA was subjected for a second round of DNase digestion and purification. The double DNase-digested RNA was stored at –80°C.

### MeRIP protocol for the detection of 5mC and m6A containing RNA

Twelve microgram of double DNase-digested nuclear RNA was sonicated using a Covaris S220 (settings: 105 Peak Power, 10.0 Duty Factor, 200 cycles per burst, and 60 s sonication time) to generate RNA fragments with an average length of 500 nt. Nine microgram of sonicated RNA was spiked with 1 ng of *in vitro* transcribed (IVT) EGFP RNA (see below), where either all cytidines are 5mC or all adenosines are m6A methylated, and the volume was adjusted to 60 μl. The RNA mixes were denatured at 70°C for 10 min and divided equally into three samples: (i) 20 μl input (stored at –80°C until cDNA synthesis), (ii) 20 μl immunoprecipitation (IP) and (iii) 20 μl beads control. The IP samples were incubated with 5 μg of antibody in 500 μl 1× MeRIP buffer (10 mM Na-Phosphate pH 7.0, 0.14 M NaCl, 0.05% Triton X-100) overnight (O/N) on a rotating wheel at 4°C. The following antibodies were used for MeRIP: α-5mC (Zymo Research, mouse monoclonal, clone 10G4) or α-m6A (Abcam, rabbit monoclonal, cat. no. ab190886). 40 μl of blocked (0.1% BSA in PBS for 2 h at 4°C) Magnetic Protein G Dynabeads (Thermo Fisher Scientific) were then added to the IP and the beads control samples, and incubated for another 2 h at 4°C. Samples were washed five consecutive times in 700 μl of fresh 1× MeRIP buffer for 10 min at room temperature (RT). After the last wash, magnetic Dynabeads were resuspended in 200 μl of Proteinase K buffer (50 mM Tris–HCl pH 8.0, 10 mM EDTA, 0.5% SDS), and 70 μg of Proteinase K (Thermo Fisher Scientific) were added for 3 h at 50°C. RNA was purified from the supernatant using the RNeasy MinElute Cleanup kit (Qiagen) and eluted in 20 μl nuclease-free water (Qiagen). For reverse transcription, equal volumes (10 μl) of input, IP and beads control were used to generate cDNA (see also Supplementary Figure S1A).

### Detection of MSR and LINE L1MdA 5′UTR RNA by directed RT-qPCR

Reverse transcription (RT) was performed using the SuperScript II Reverse Transcriptase kit (Thermo Fisher Scientific), 0.5 mM of dNTP mix (Thermo Fisher Scientific) and 200 ng of random hexamer primers (Thermo Fisher Scientific). The cDNA was diluted 10x in water and stored at –20°C. 5 μl (37.5 ng) of the 10x diluted cDNA was mixed with 2× SYBR Select Master Mix (Thermo Fisher Scientific) and 200 nM of target-specific forward and reverse primers (Sigma-Aldrich) in a total volume of 10 μl. qPCR was performed with the QuantStudio 6 Flex machine (Applied Biosystems) with an annealing temperature of 60°C in a program using 40 cycles. Cycle threshold (Ct) values were used to calculate normalized expression (ΔΔCt method) and enrichment over input was calculated using the ΔCt method.

### Capture of MSR and LINE L1MdA 5′UTR transcripts with biotinylated DNA probes

We cultivated approximately 2.4 × 10^9^ (240 maxi dishes) J1 WT ES cells (54) for nuclear RNA isolation. The nuclear RNA was pooled to a total of 5.7 mg, double DNase I digested and sonicated. For the capture of MSR transcripts, 4.7 mg of this processed RNA was mixed with four biotinylated DNA probes (400 pmol each) that are complementary to the MSR reverse sequence. For capture of LINE L1MdA 5′UTR transcripts, 1 mg of this processed RNA was mixed with three biotinylated DNA probes (400 pmoles each) that are complementary to the LINE L1MdA 5′UTR forward sequence (see Supplementary Figure S2A). The mixture of RNA and biotinylated DNA probes was distributed into 10 Eppendorf tubes (100 μl each), denatured for 3 min at 90°C in 5× SSC buffer, cooled down on ice and hybridized for 10 min at 65°C. The hybridized samples were then kept at RT. 60 μl of washed (three times 1x BW buffer and one time 5× SSC buffer) MyOne Streptavidin C1 Dynabeads (Thermo Fisher Scientific) were added to each of the hybridized samples and incubated at 600 rpm (Thermomixer Eppendorf) for 30 min at RT. The Dynabeads were washed (once with 1× SSC buffer and three times with 0.1× SSC buffer), resuspended in 25 μl of nuclease-free water (Qiagen) and incubated for 3 min at 75°C in order to release the RNA. Released RNA from either the MSR-enriched samples or from the LINE L1MdA 5′UTR-enriched samples were then combined in one tube (final volume of 100 μl) and processed for a second round of capture with the biotinylated DNA probes. The 2x captured RNA samples were digested with DNase I for 30 min at 37°C, and the RNA was precipitated with 3 volumes of 100% ethanol, 0.5 M ammonium acetate, 15 μg of GlycoBlue (Thermo Fisher Scientific) O/N at –20°C, washed with 70% ethanol, resuspended in nuclease-free water (Qiagen) and stored at –80°C. The RNA concentration was measured using the Quant-iT Ribo-Green RNA Assay (Thermo Fisher Scientific) on a Nanodrop 3300 and the RNA quality was monitored using a Bioanalyzer RNA 6000 Pico Kit (Agilent). An aliquot (ca. 5ng) of the 2× captured RNA samples was taken for MiSeq sequencing to control for the purity of the enrichment (see Supplementary methods and Supplementary Figure S2B). The final amount of 2× captured RNA that was processed by LC–MS/MS was 114 ng for MSR-reverse and 183 ng for LINE L1MdA 5′UTR-forward RNA.

### LC–MS/MS to detect RNA modifications in captured RNA

Liquid chromatography - tandem mass spectrometry analysis was performed following the protocol for analysis of RNA modifications (55). MSR-reverse (114 ng) and LINE L1MdA 5′UTR-forward (183 ng) 2× captured RNA samples were digested with nuclease P1 (Sigma-Aldrich) and snake venom phosphodiesterase (SVPD), and subsequently dephosphorylated with alkaline phosphatase (Thermo Fisher Scientific). Single nucleosides were then separated using a RP-18 high performance liquid chromatography (Agilent) with a gradient from 10–40% ACN. Separated nucleosides were subjected to ionization and mass analysis using the Agilent 6460 Triple Quadrupole mass spectrometer. Modified nucleosides were identified by retention time and molecular weight and compared to internal standards.

### Generation of MSR and LINE L1MdA 5′UTR RNA by *in vitro* transcription

PCR products were amplified from the pSAT-MSR-1-repeat or pEX-L1MdA-1-repeat plasmids (7,56) using primers containing the T7 promoter. MSR-sense, MSR-antisense and LINE L1MdA 5′UTR sense and antisense transcripts were then generated by *in vitro* transcription (IVT) with T7 RNA polymerase (MEGAScript T7 Transcription Kit, Thermo Fisher Scientific). For mutant IVT MSR and LINE L1MdA 5′UTR transcripts lacking either all adenosines or containing the RRACHmut motifs, corresponding DNA templates with a flanking T7 promoter sequence were purchased from Integrated DNA Technologies (IDT). The EGFP IVT transcript was generated from the pCR4-TOPO-EGFP-S1 plasmid (Jenuwein lab) that was linearized with NotI prior to *in vitro* transcription with T3 RNA polymerase. For the EGFP IVT transcripts containing either 5mC or m6A, CTP was replaced by 5mCTP or ATP was replaced by m6ATP (both TriLink Biotechnologies) in the *in vitro* transcription reactions. The IVT transcripts were purified using the RNeasy MinElute Cleanup kit (Qiagen) and their quality was assessed on a Bioanalyzer RNA 6000 Nano Kit (Agilent).

### RNA methylation assay with recombinant METTL3/METTL14 complex

The RNA methyltransferase assay was adapted from a published protocol (38). 5 μM of IVT transcripts were reacted with 225nM of recombinant human METTL3/METTL4 (Active Motif, cat. no. 31570) in a buffer (15 mM HEPES pH 7.3, 50 mM NaCl, 1 mM MgCl_2_, 1 mM dithiothreitol (DTT), 50 mM KCl, 4% glycerol) containing 40 μM *S*-adenosyl-l- methionine (SAM) plus 1.1 μCi *S*-[methyl-^3^H]-SAM (Perkin Elmer) for 3 h at 30°C. The RNA was then purified with an RNA Clean & Concentrator kit (Zymo Research) and added to Ultima Gold scintillation fluid (Perkin Elmer), and the methyl-^3^H incorporation (counts per minute, CPM) were measured with the Tri-Carb 2910 TR (Perkin Elmer).

### Generation of *Mettl14*-mutant ES cells (A10 clone)

Single guide RNAs (sgRNA) targeting the MT-A70 domain of either Mettl3 and Mettl14 were designed using the CRISPR design online tool (57) and cloned into the Cas9 expression vector pSpCas9(BB)-2A-Puro (pX459, Addgene). WT ES cells were grown in a six-well plate and cells in one well were co-transfected at ∼80% confluency with the pX459-Mettl3ex5 (2.5 μg) and pX459-Mettl14ex10 (2.5 μg) plasmids using Xfect Transfection Reagent (Takara) to obtain double *Mettl3*/*Mettl14* CRISPR mutants. After 48 h, transfected cells were selected with 1.25 μg/ml puromycin for two days. Surviving cells where then collected (mixed population of cells) and an aliquot of cells was used to perform a surveyor assay (57), while the remaining culture was re-seeded in maxi dishes at 1:1000, 1:2000, 1:5000 dilutions to obtain single clones. After 1 week, 192 clones were manually picked and split into 96-well plates. Cells from one half of the clones were lysed in ‘tail/ear’ lysis buffer (10 mM KCl, 20 mM Tris–HCl pH 8.0, 10 mM (NH_4_)_2_SO_4_, 1 mM EDTA, 0.1% Triton X-100) and 0.1 mg/ml of Proteinase K (Thermo Fisher Scientific) for minimum 4 h at 50°C, followed by a 10 min 95°C inactivation of Proteinase K. Lysates, 10x diluted in water, were directly used for PCR (Terra Taq polymerase (Takara)) with primers amplifying exon 5 of *Mettl3* and exon 10 of *Mettl14*. PCR amplicons were sequenced by Sanger sequencing. Out of 192 clones we identified only one clone (A10 clone) that is homozygous mutant for *Mettl14* (see Supplementary Figure S3). The A10 clone contains an intact MT-A70 domain of *Mettl3*.

### HiSeq RNA sequencing of m6A enriched nuclear RNA

Three times 10 μg of double DNase-digested nuclear RNA of WT/RBC or *Mettl14*/*Mettl3* KO ES cells was processed for m6A MeRIP as described above. The resulting material from input and IP samples of the three MeRIP experiments (1 biological replicate) was combined and concentrated with a Concentrator 5301 (Eppendorf) to a final volume of 12 μl. The quality of the RNA was controlled by Fragment Analyzer (Agilent) and the concentration was measured using the Qubit RNA High Sensitivity assay (Thermo Fisher Scientific). Between 10 and 100 ng of the IP samples were used to prepare ribosomal RNA-depleted cDNA libraries (random hexamers) using the TruSeq Stranded Total RNA Library Prep Gold protocol (Illumina), but without the fragmentation step. Paired-end, 75 bp reads were generated with the HiSeq3000 sequencer (Illumina) with a coverage of 30 million reads per sample. Two biological replicates of m6A MeRIP enrichment were analyzed.

### Bioinformatic analysis of distinct repeat classes in m6A enriched RNA

Unprocessed reads were trimmed using cutadapt (58) version 1.8.1. Reads were then aligned to the GRCm38 genome from Ensembl using STAR (59) version 2.6.0c, with settings (–sjdbOverhang 100 –winAnchorMultimapNmax 200 –outFilterMultimapNmax 100) that identify repeat reads as recommended by TEtranscripts (60). GTF files of repeat annotations were generated from the RepeatMasker (61) (http://www.repeatmasker.org) obtained from UCSC genome database (62). Repeat reads were quantified using TEtranscripts version 2.0.3 with settings (–mode multi –minread 1 -i 10 –padj 0.05) and differentially enriched reads from distinct repeat elements were analyzed in R with the DESeq2 (63) package version 1.22.2. Data visualization was performed in R version 3.5.0 using the ggplot2 (64) package. For the analysis of non-repeat transcripts, reads were aligned to the GRCm38 genome from Ensembl using STAR (59) version 2.6.0c with settings (–sjdbOverhang 100 –outFilterMultimapNmax 1 –alignIntronMax 1). m6A-enriched peaks were identified by MeTDiff peak calling software (65) with input samples serving as control. Peaks with a FDR ≤ 0.05 were annotated using ChiPseeker software (66) version 1.18.0. Motif analysis was performed *de novo* with HOMER (67) using significantly enriched peaks (FDR ≤ 0.05) at 3′UTR regions and the script findMotifsGenome.pl with parameters ‘mm10-rna’. Differential expression of m6A-enriched reads between WT26 versus *Mettl14* KO and RBC versus *Mettl3* KO samples was detected with MeTDiff. The differential m6A peaks (FDR ≤ 0.05) were annotated by ChIPseeker. Pathway enrichment analysis was performed on the differential m6A peaks using ReactomePA package (68) version 1.24.0.

### Purification of RNA from cytoplasmic, nucleoplasmic and chromatin fractions

RNA preparations from sub-cellular fractions were done according to published protocols (53) and with the following adaptations. 2 × 10^7^ ES cells were pelleted, washed and resuspended in 1 ml of ice-cold buffer A (0.1% NP-40, 10 mM HEPES pH 7.9, 5 mM MgCl_2_ and 0.25 M sucrose) with an 18G needle (10 strokes). The lysate was rotated for 10 min at 4°C and centrifuged at 6000 g for 10 min at 4°C. The supernatant (cytoplasmic fraction) was aspirated, mixed with 1 ml of TRIreagent (Sigma-Aldrich) and stored at –80°C. The nuclear pellet was resuspended in 1 ml of ice-cold buffer B(0.4) (20 mM HEPES pH 7.9, 1 mM MgCl_2_, 0.1 mM EDTA, 0.4 M NaCl and 25% glycerol) with an 18G needle (10 strokes). The lysate was rotated for 30 min at 4°C and centrifuged at 10 000 g for 10 min at 4°C. The supernatant (nucleoplasmic fraction) was aspirated, mixed with 1 ml of TRIreagent (Sigma-Aldrich) and stored at –80°C. The chromatin pellet was resuspended in 1 ml of ice-cold buffer B(2.0) (20 mM HEPES pH 7.9, 1 mM MgCl_2_, 0.1 mM EDTA, 2 M NaCl and 25% glycerol) with an 18 G needle (10 strokes). The lysate was rotated for 30 min at 4°C, sonicated with 10 pulses (30 s ON, 30 s OFF) using a Bioruptor (Diagenode) until the viscosity was reduced and then mixed with 1 ml of TRIreagent (Sigma-Aldrich). The subsequent purification of TRIreagent isolated RNA from the cytoplasmic, nucleoplasmic and chromatin fractions and double DNase digestion was done as described above.

### Extraction of chromatin-associated nucleic acids for RNA:DNA hybrid detection

Extraction of chromatin-associated nucleic acids (NA) for RNA:DNA hybrid detection was performed with phenol/chloroform. This allowed for a more robust RDIP signal as compared with material that was isolated by TRIreagent (data not shown). A sonicated chromatin lysate was prepared from 1 × 10^7^ ES cells, which was then subjected to total NA isolation using phenol/chloroform (69). The phenol/chloroform extracted NA were transferred to a microTUBE (Covaris) and again sonicated using a Covaris S220 with the following settings: 105 Peak Power, 5.0 Duty Factor, 200 cycles per burst, and 80 s sonication time. The sonicated NA were run on a 1% agarose gel to verify that the NA fragments were between 200 and 1000 bp. Sonicated NA were stored at –80°C.

### RNase H digestion (37°C) of chromatin-associated nucleic acids

To perform RNase H digestion, we followed a protocol that allows comparison of RNase H treated and untreated NA at the same temperature (7). 10 μg of chromatin-associated, phenol/chloroform-isolated NA were incubated for 2 h at 37°C with 13 U of RNase H (NEB) in 1× buffer (NEB) in a total volume of 30 μl. The untreated (i.e. without RNase H) control (10 μg) was also incubated for 2 h at 37°C in 1× NEB buffer. RNase H treated and untreated samples were then double DNase I digested and processed for directed RT-qPCR.

### Expression and purification of recombinant HBD(RNaseH1)

The hybrid binding domain (HBD) sequence, spanning amino acids 27–76 from mouse RNase H1 (70), and the nuclear localization sequence (NLS) PKKKRKV were cloned with the Gateway system (Thermo Fisher Scientific) to generate 6xHis-MBP-NLS-HBD(H1)-eGFP and 6xHis-MBP-NLS-eGFP control constructs. All constructs were verified by sequencing. The 6xHis-MBP fusion proteins were expressed in Rosetta *Escherichia coli* strain as described (7), with the following adjustments. After IPTG induction, the bacterial lysate was sonicated using a Sonoplus sonicator (Bandelin) (50% power, 5 times: 15 s ON, 45 s OFF) and centrifuged twice for 30 min at 4°C. The supernatants were incubated with 3 μl of Pierce Universal Nuclease (Thermo Fisher Scientific) for 30 min at RT and cleared using a syringe and a 0.45 μm Whatman filter (GE Healthcare). Cleared lysates were purified using a FPLC (AKTAexplorer) with a MBPTrap HP 1 ml column (GE Healthcare). To cleave the 6xHis-MBP-tag, the purified proteins were incubated with 15 μl (150 U) of AcTEV protease (Thermo Fisher Scientific) O/N at 4°C in 1x TEV Buffer (Thermo Fisher Scientific). The NLS-HBD(H1)-eGFP and NLS-eGFP proteins were separated with a HisTrap HP 1 ml column (GE Healthcare). Purified proteins were aliquoted at 1 μg/μl, snap-frozen in liquid nitrogen and stored at -80°C. Residual bacterial nucleic acids (primarily RNA) are associated with purified NLS-HBD(H1)-eGFP. To examine whether bacterial NA could affect the affinity of NLS-HBD(H1)-eGFP in RDIP, we cleared the recombinant protein with RNase A (Thermo Fisher Scientific) and Pierce Universal Nuclease (Thermo Fisher Scientific). This RNase A/Nuclease treatment did not significantly improve the intrinsic RNA:DNA hybrid binding potential of NLS-HBD(H1)-eGFP, but may be required if the NLS-HBD(H1)-eGFP reagent is used for genome-wide RDIP analyses (data not shown).

### RDIP detection of RNA:DNA hybrids using HBD(RNaseH1): RNA amplification

For the optimization of a RNA:DNA immunoprecipitation (RDIP) protocol to detect MSR and LINE L1MdA 5′UTR repeat sequences, we compared TRIreagent-isolated NA and phenol/chloroform-isolated NA and incubation with RNase H at 37°C or at 4°C. We obtained the most reproducible results with phenol/chloroform-isolated NA and RNase H incubation at 4°C. In addition, RNA:DNA hybrid detection by recombinant HBD(H1)-eGFP is more efficient at 4°C. We therefore used an RNase H enzyme (Roche) that has robust activity at 4°C.

2.1 μg of chromatin-associated, phenol/chloroform-isolated NA were incubated for 2 h at 4°C with 13 U of RNase H (Roche) in 1x buffer (20 mM HEPES–KOH, 50 mM KCl, 10 mM MgCl_2_ and 1 mM DTT) in a total volume of 30 μl. The untreated control (2.1 μg) was also incubated for 2 h at 4°C in 1× buffer in a volume of 30 μl. RNase H-treated and untreated samples were then diluted to a volume of 42 μl and the samples were immediately subjected to RDIP with the HBD(H1)-eGFP (20 μl) or with the eGFP control (20 μl). 2 μl were saved as input.

The RNase H treated or the untreated material (20 μl each) was combined with 10 μl of GFP-Trap magnetic beads (Chromotek) that had been coupled to either 1 μg of recombinant HBD(H1)-eGFP or eGFP. Samples were then incubated for 90 min at 4°C in 2× EMSA buffer (40 mM Tris pH 8.0, 300 mM NaCl, 10 mM MgCl_2_, 25% glycerol, 0.1% Triton X-100). The beads–protein–NA complexes were sequentially washed with for 5 min at RT in high salt buffer (50 mM Tris–HCl pH 7.5, 0.5 M NaCl, 5 mM EDTA pH 8.0, 1% Triton X-100 and 0.1% Na-deoxycholate), low salt buffer (50 mM Tris–HCl pH 7.5, 0.14 M NaCl, 5 mM EDTA pH 8.0, 1% Triton X-100, and 0.1% Na-deoxycholate), IP-washing buffer (10 mM Tris–HCl pH 8.0, 0.25 M LiCl, 0.5% NP-40, 0.5% Na-deoxycholate and 1 mM EDTA) and 10– TE buffer. After the last wash, the beads-protein-NA complexes were resuspended in 100 μl of RDIP-elution buffer (50 mM Tris–HCl pH 8.0, 10 mM EDTA and 2% SDS) and incubated for 15 min at 65°C in a thermomixer (600 rpm). Input samples (2 μl, 10%), that were stored on ice, were combined with 98 μl RDIP-elution buffer and also incubated for 15 min at 65°C in a thermomixer (600 rpm).

For the PCR detection of enriched MSR RNA:DNA hybrids, we compared DNA amplification vs. RNA amplification of the eluted material and observed a more robust and reproducible signal by using RNA amplification (see Supplementary Figure S11). Therefore, RNA was isolated with 0.5 ml of TRIreagent (Sigma-Aldrich) and MaXtract High Density 1.5 ml columns (Qiagen) and double DNase I digested. Equal volumes (10 μl) of input, HBD(H1)-eGFP and eGFP-enriched samples were processed with the SuperScript II Reverse Transcriptase kit (Thermo Fisher Scientific) using random hexamers. 1 μl of undiluted cDNA was used for qPCR with the QuantStudio 6 Flex machine (Applied Biosystems) with an annealing temperature of 60°C in a program using 40 cycles.

### RDIP detection of RNA:DNA hybrids using the S9.6 antibody: RNA amplification

One microgram of chromatin-associated, phenol/chloroform-isolated NA were either treated with RNase H (Roche) or untreated for 2 h at 4°C. This material was then incubated with 25 μl of magnetic Protein G Dynabeads (Thermo Fisher Scientific) that had been coupled to 1 μg of the monoclonal S9.6 antibody (Merck Millipore, cat. no. MABE 1095) O/N at 4°C in a rotating wheel. The next day, the bead-protein-NA complexes were washed three times for 5 min at RT with ChIP-wash buffer (0.1% SDS, 1% Triton X-100, 2mM EDTA pH 8.0, 150 mM NaCl, 20 mM Tris–HCl pH 8.0 and protease inhibitors (Roche)) and once for 10 min at RT with the ChIP-final wash buffer (0.1% SDS, 1% Triton X-100, 2 mM EDTA pH 8.0, 500 mM NaCl, 20 mM Tris–HCl pH 8.0, and protease inhibitors (Roche)). The bead-protein-NA complexes were then resuspended in 100 μl of RDIP-elution buffer and processed for detection of MSR and LINE L1MdA 5′UTR RNA:DNA hybrids by RNA amplification as described above.

### Electrophoretic mobility shift assay (EMSA)

EMSA was performed as described (7). RNA:DNA hybrids were generated with 5′-Cy5 labeled or unlabeled 35 nt RNA and DNA oligonucleotides (Sigma-Aldrich, BioSynthesis) spanning a sequence from subrepeat 2 of the MSR (see Supplementary Table S1). In addition, 5mC and m6A modified RNA oligonucleotides (Sigma-Aldrich, BioSynthesis) were also used. Equimolar amounts of RNA and DNA oligonucleotides (0.5 μM each) were mixed in 1× MES buffer (2-(N-morpholino)ethanesulfonic acid 0.5 M, pH 7.0), denatured for 3 min at 90°C, annealed for 30 min at 37°C, cooled for 1 h at 4°C and then stored at –20°C. For EMSA, 20 nM of RNA:DNA hybrids were incubated with increasing concentrations (e.g. 125 nM to 0.5 μM) of recombinant HBD(H1)-eGFP or eGFP in 1x binding buffer (20 mM Tris–HCl pH 8.0, 150 mM NaCl, 5 mM MgCl_2_, 12.5% glycerol, 0.05% Triton X-100 and 1 mM DTT) for 1 h at 4°C. Reaction products were resolved on a 4% polyacrylamide (60:1) gel (25 mM Tris–HCl, 250 mM glycine, 5% glycerol, 0.075% ammonium persulfate and 0.05% TEMED) in running buffer (12.5 mM Tris–HCl and 100 mM glycine) and the Cy5 signal was detected using a Typhoon FLA 9500 fluorescence scanner (GE Healthcare).

### Statistical analysis

Where indicated, the data are presented as mean values ± SD. Statistical significance between groups was determined using paired or unpaired two-tailed *t*-test or the Wald test. *P*-values ≤0.05 were considered to be statistically significant.

## RESULTS

### Heterochromatic RNA has abundant m6A RNA methylation

**Figure 1.**
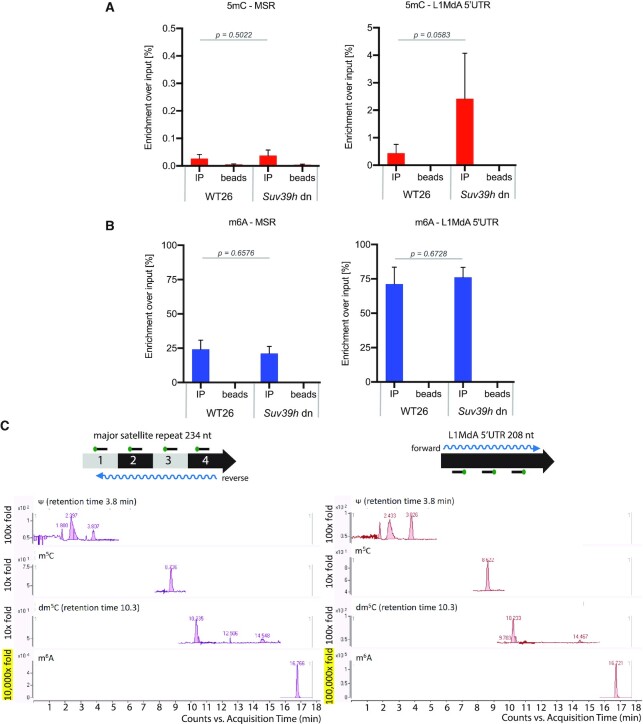
Heterochromatic RNA has abundant m6A RNA methylation. (**A**) MeRIP analysis of 5mC modified MSR (left) and LINE L1MdA 5′UTR (right) transcripts in WT26 and *Suv39h* dn ES cells. Double DNase digested nuclear RNA was probed with an α-5mC antibody and enriched transcripts were analyzed by RT-qPCR. Enrichment is calculated as a percentage of 5mC-positive transcripts over the total amount of input transcripts. The data represent the mean ± SD from *n* = 3 biological replicates. (**B**) The same as in (A), but the MeRIP analysis was performed with an α-m6A antibody. For (A) and (B), statistical significance was determined by unpaired two-tailed t-test and the *P*-values are indicated. (**C**) LC-MS/MS chromatogram of Ψ, m^5^C, dm^5^C and m^6^A in MSR-enriched (left) and LINE L1MdA 5′UTR-enriched (right) RNA samples. Capture of MSR reverse strand and of LINE L1MdA 5′UTR forward strand from nuclear RNA of J1 WT ES cells was performed with biotinylated DNA probes (see scheme above the chromatograms). Indicated retention times for Ψ and dm^5^C specify the signal for these modifications over other detected peaks. Only background levels (10-fold signal) were observed for m^5^C RNA, whereas high (10 000-fold signal) to very high (100 000-fold signal) levels were detected for m^6^A RNA, as indicated on the left of the chromatograms. *n* = 1.

To identify RNA modifications on hetRNA, we optimized a protocol for MeRIP (methylated RNA immunoprecipitation) coupled to RT-qPCR (see Supplementary Figure S1A and Materials and methods). We used antibodies recognizing either 5mC or m6A modifications in nuclear RNA of mouse embryonic stem cells (ES). For this, we focused on wild type 26 (WT26) and *Suv39h* double null (dn) ES cells, as there is more transcripts of MSR and LINE L1MdA 5′UTR in *Suv39h* dn ES cells (71). With the α-5mC antibody, we observed a very low enrichment of around 0.02% for 5mC MSR RNA in input RNA from WT26 cells (Figure 1A, left). This signal was was not considerably increased in RNA samples from *Suv39h* dn ES cells. For the LINE L1MdA 5′UTR transcripts, we observed a greater 5mC RNA enrichment and this enrichment was further enhanced (although not within a statistically significant range) in RNA samples from *Suv39h* dn ES cells to the level of ∼2% of input RNA (Figure 1A, right).

In comparison to 5mC levels, we observed 20% enrichment over input for m6A MSR RNA in WT26 cells (Figure 1B, left) and a very high level, 70% over input, of m6A RNA for LINE L1MdA 5′UTR transcripts (Figure 1B, right). The m6A MSR and LINE L1MdA 5′UTR RNA methylation levels did not change between WT26 and *Suv39h* dn cells. To demonstrate the efficiency of our MeRIP protocol, we also analyzed m6A-positive mRNA for *Klf4*, *Sox2* or *Nanog* (45) (Supplementary Figure S1B, left) and used a fully m6A-methylated EGFP *in vitro* transcript as a spike-in control (Supplementary Figure S1B, right). Together, our MeRIP analysis indicates only background levels of 5mC but an abundance of m6A methylation on hetRNA. We conclude that around 20% of MSR transcripts and 70% of LINE L1MdA 5′UTR transcripts are m6A modified.

To analyse hetRNA modification by an antibody-independent approach, we next enriched hetRNA. For this, we captured MSR forward, MSR reverse and LINE L1MdA 5′UTR forward transcripts with complementary biotinylated DNA probes from bulk nuclear RNA of WT ES cells (see Supplementary Figure S2A and Materials and methods). Samples were controlled for specific enrichment prior to liquid chromatography–tandem mass spectrometry (LC–MS/MS). MiSeq RNA sequencing shows that in the MSR-reverse RNA-enriched sample, the majority of the reads mapped to the consensus sequence of major satellite repeats (GSAT_MM), and for the LINE L1MdA 5′UTR-forward RNA-enriched sample, the majority of the reads mapped to two subtypes of the L1MdA subfamily (L1MdA_I and L1MdA_III, Supplementary Figure S2B). The MSR-forward RNA-enriched sample was still containing residual 28S rRNA (data not shown) and was therefore not used for LC–MS/MS.

LC–MS/MS detected RNA 5-methylcytidine (here abbreviated as m^5^C) signal at a background level (10-fold) for both, MSR reverse-enriched (Figure 1C, left) and LINE L1MdA 5′UTR forward-enriched (Figure 1C, right) samples, at a similar level to the DNA 5-methyldeoxycytidine (dm^5^C). The existence of DNA 5-methyldeoxycytidine (dm^5^C) suggests some contamination of DNA coming either from genomic DNA or DNA oligonucleotides used for hybridization (72). In contrast, the methyl-6-adenosine (m^6^A) was detected at 10,000-fold higher signal for MSR reverse-enriched transcripts (Figure 1C, left) and 100 000-fold higher signal for LINE L1MdA 5′UTR forward-enriched transcripts (Figure 1C, right).

The combined data from the MeRIP and mass spectrometry analyses therefore reveal a significant enrichment of m6A RNA, as compared to only background levels for 5mC RNA, for MSR and LINE L1MdA 5′UTR repeat transcripts.

### MSR-sense transcripts are preferred substrates for the METTL3/14 methyltransferase complex

The m6A modification found in RNA Pol II transcripts is mostly deposited by the Mettl3/Mettl14 methyltransferase complex (45,46). We next asked if hetRNA were substrates for Mettl3/Mettl14 in an *in vitro* methyltransferase assay. The Mettl3/Mettl14 is highly conserved between human and mouse, and we used a commercially available human recombinant METTL3/METTL14 complex. *In vitro* transcribed single stranded RNA spanning the sequence of one repeat of MSR or of 5′UTR of LINE L1MdA, in the sense (S) or antisense (AS) orientation, were incubated with METTL3/METTL14 and S-[methyl-3H]-(SAM), and incorporation of m6A into RNA was measured by scintillation counting.

**Figure 2.**
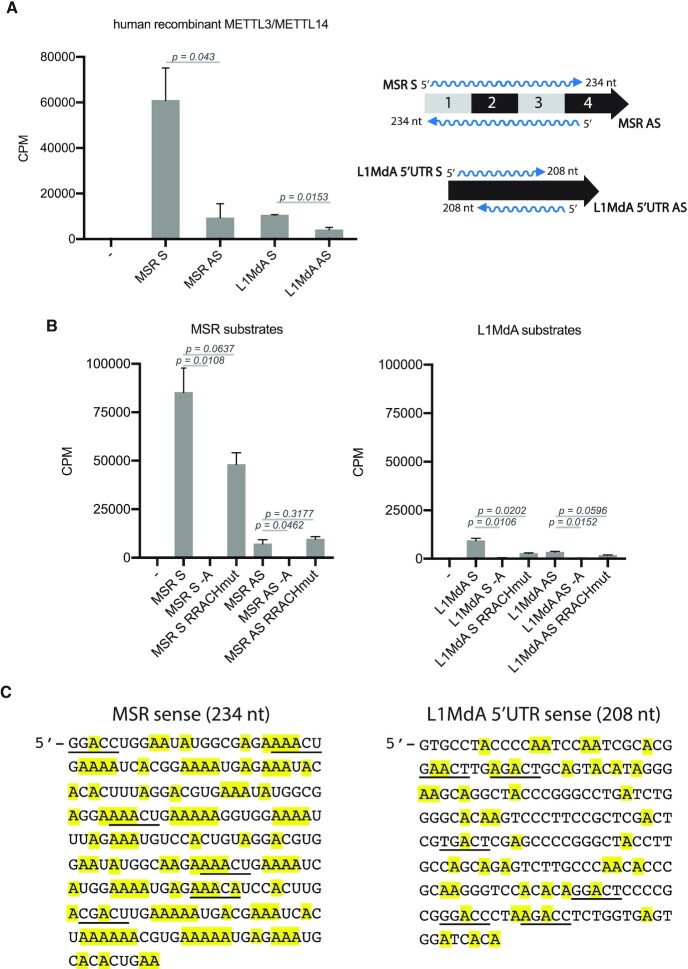
MSR forward transcripts are preferred substrates for the METTL3/14 methyltransferase complex. (**A**) Histogram showing the results of an *in vitro* RNA methyltransferase assay with human recombinant METTL3/METTL14 complex and *in vitro* transcribed single stranded RNA for MSR sense (S) or antisense (AS) and LINE L1MdA 5′UTR sense (S) or antisense (AS) transcripts. Control reaction was performed without RNA (–). Incorporation of *S*-[methyl-^3^H]-SAM into the RNA substrates was measured as counts per minute (CPM). The data represent the mean ± SD from *n* = 3 technical replicates. (**B**) The same as in (A), but using additional RNA substrates in which the middle adenosine of the RRACH motif was mutated to uridine (RRACHmut) or where all adenosines were replaced by uridines (–A). For (A) and (B), statistical significance was determined by unpaired two-tailed t-test and the *P*-values are indicated. (**C**) DNA consensus sequences (sense strand) of one MSR repeat unit (234 nt) (left) and of one LINE L1MdA 5′UTR repeat unit (208 nt) (right). RRACH motifs that are present in these sequences are underlined. Adenosines are highlighted in yellow.

The METTL3/METTL14 complex methylated all RNA substrates (≥4000 counts per minute, CPM) when compared to no substrate controls (Figure 2A). Notably, there was a very high activity (∼60 000 CPM) towards the MSR-sense RNA but not the MSR-antisense RNA. The LINE L1MdA 5′UTR sense and antisense transcripts were only observed as weak substrates. These results indicate that the MSR-sense (forward) RNA is a preferred substrate for the METTL3/METTL14 complex *in vitro*.

The m6A RNA methyltransferase complex was described to recognize and methylate the adenosine found in the ‘RRACH’ sequence motif (where R indicates adenosine or guanosine, and H indicates adenosine, cytidine, or uridine) (34,35). Although the MSR sense and LINE L1MdA 5′UTR sense RNA consensus sequences each contain six RACH motifs, the MSR sense RNA has a much higher A content (46% A-rich) (see Figure 2C). We generated *in vitro* transcripts that have the RRACH motif mutated to RRUCH sequence (RRACHmut) or where all adenosines found in the MSR and LINE L1MdA 5′UTR RNA sequences were replaced with uridines (‘minus A’, –A). We examined these mutated RNA sequences as substrates for the METTL3/METTL14 complex. m6A methylation was decreased by half when comparing the MSR sense RRACHmut RNA to MSR-sense RNA. By contrast, the MSR-antisense RRACHmut RNA did not show any difference when compared to the MSR-antisense RNA (<20% A-rich) (Figure 2B, left). For the LINE L1MdA 5′UTR, both sense and antisense transcripts containing the RRACHmut motifs were also observed to be less m6A methylated by the METTL3/ METTL14 complex (Figure 2B, right). All transcripts without adenosines resulted in background signals similar to the no-substrate control (Figure 2B).

The MSR sense transcript contains six RRACH motifs (underlined, Figure 2C, left), compared to only one RRACH motif in the MSR-antisense transcript (not shown). Both the sense and antisense MSR RNA largely display single-stranded regions in their secondary structure. Although the LINE-sense transcript also contains six RRACH motifs (underlined, Figure 2C, right), the secondary structure of the LINE L1MdA 5′UTR transcripts is distinct and primarily folds into dsRNA (7). While RNA secondary structures could contribute to substrate specificity of the Mettl3/Mettl14 complex, the data are most consistent with the overall A content of target RNA to direct high levels of m6A RNA methylation. We conclude that the METTL3/METTL14 complex can methylate LINE L1MdA 5′UTR and MSR RNA sequences *in vitro*.

### *Mettl14* and *Mettl3* mutant ES cells have reduced levels of m6A hetRNA methylation

We then examined if the Mettl3/Mettl14 complex methylates hetRNA in mouse ES cells. We used the CRISPR/Cas9 system in WT26 ES cells (129/Sv × C57Bl6 background) to generate knock-out (KO) cell lines. We chose to target the catalytically active MT-A70 domain of Mettl3 and the degenerated MT-A70 domain of Mettl14, which has been shown to be important for the dimerization of both proteins (36).

Single guide RNAs (sgRNA) for MT-A70 of *Mettl3* and MT-A70 of *Mettl14* were co-transfected to obtain double CRISPR/Cas9 *Mettl3/Mettl14* KO in WT26 cells. Although no double KO was obtained, a single *Mettl14* KO clone (A10 clone) was derived. It has a 4 bp homozygous deletion that causes a frame shift and generates a premature termination codon, resulting in a truncated 311 aa long Mettl14 (Supplementary Figure S3). The A10 clone contains an intact MT-A70 domain of the *Mettl3* gene. An additional approach for single CRISPR/Cas9 gene disruptions of *Mettl3* or *Mettl14* was performed, however unsuccessfully. We therefore focused on the A10 clone and obtained *Mettl3* KO ES cells from a different study (73). The *Mettl3* KO cells (2c4d clone) were derived from engineered WT ES cells (129/Sv × C57Bl6 background), termed RBC (Rosa26 BirA-V5, Cre-ERT2 recombinase) (73).

**Figure 3.**
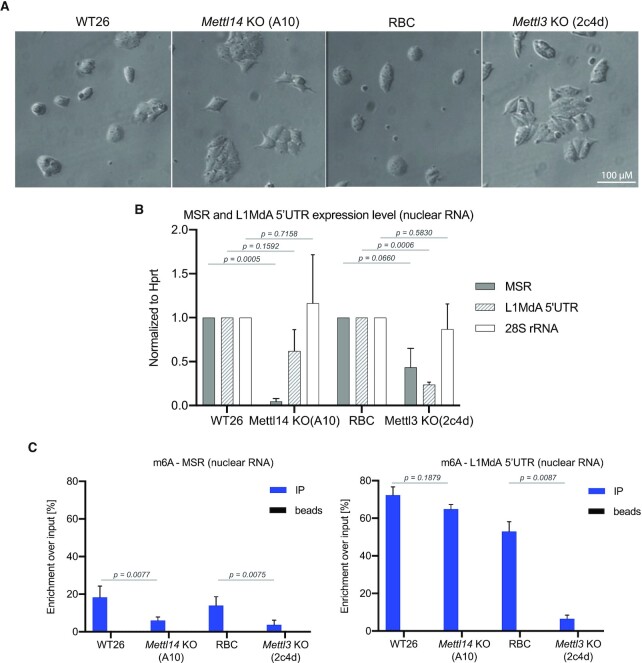
*Mettl14* and *Mettl3* mutant ES cells display reduced levels of MSR m6A RNA methylation. (**A**) Morphology of WT26, *Mettl14* KO (clone A10), RBC and *Mettl3* KO (clone 2c4d) ES cells under bright-field microscopy. Scale bar = 100 μM. (**B**) Histogram showing the expression level of MSR (grey bar), LINE L1MdA 5′UTR (hatched bar) and 28S rRNA (white bar) transcripts in WT26, *Mettl14* KO (clone A10), RBC and *Mettl3* KO (clone 2c4d) ES cells. Expression was analyzed in nuclear RNA by RT-qPCR and normalized to the expression of *Hprt* in WT26 cells. The data represent the mean ± SD from n = 3 biological replicates. (**C**) MeRIP analysis of m6A-enriched MSR (left) and LINE L1MdA 5′UTR (right) transcripts in nuclear RNA from WT26, *Mettl14* KO, RBC and *Mettl3* KO ES cells. Enrichment is calculated as the percentage of m6A-positive transcripts over the total amount of input transcripts. The data represent the mean ± SD from *n* = 4 biological replicates. For (B) and (C), statistical significance was determined by unpaired two-tailed t-test and the *P*-values are indicated.

Both *Mettl14* KO and *Mettl3* KO cells display a similar morphological change: the loss of ES cell-like colonies and a more flattened cell shape (Figure 3A). HiSeq RNA sequencing of nuclear RNA (see Supplementary methods) indicated that *Mettl14* KO and *Mettl3* KO ES cells display a comparable dysregulation of genes (on average 450 genes up-regulated and 530 genes down-regulated) (Supplementary Figure S4A). Intriguingly, gene ontology analysis reveals alteration of pathways in neuronal differentiation (for both *Mettl14* KO and *Mettl3* KO) and in signal transduction (*Mettl14* KO) or in cell migration (*Mettl3* KO) (Supplementary Figure S4B). No pathway changes related to RNA transcription, elongation, splicing or translation are detected. This is important to examine possible indirect effects on m6A RNA methylation that may arise from altered transcriptional activity or impaired RNA PolII function, as has been shown for nascent mRNA transcripts (13,74). However, in contrast to *bona fide* mRNA, MSR repeat transcripts largely lack poly(A) tails and do not have a 5′ cap (7) and therefore may have a less stringent transcriptional control. We also generated heatmaps for expression of genes that encode core components of the transcription machinery, m6A RNA methylation and heterochromatin formation (Supplementary Figure S5). We have not observed major changes in these pathways and only very few genes are altered within a statistically significant range (*P* < 0.05). In addition, there are also no significant alterations in the expression of *RNaseH1* or *RNaseH2* genes. Although we have not analyzed transcription rates directly, these data suggest that there are no major differences for gene pathways involved in transcriptional regulation or heterochromatin formation between WT26/*Mettl14* and RBC/*Mettl3* ES cells.

We performed RT-qPCR to assess the expression level of hetRNA in *Mettl14* KO and *Mettl3* KO cells. A 10-fold reduction in the expression level of MSR transcripts and a 2-fold reduction for LINE L1MdA 5′UTR transcripts was observed in the *Mettl14* KO in comparison to WT26 (Figure 3B). For the *Mettl3* KO cells, expression of MSR and LINE L1MdA 5′UTR transcripts were also decreased by 2-fold (MSR) and 5-fold (LINE L1MdA 5′UTR) when compared to RBC controls (Figure 3B). We did not observe significant changes in the expression of 28S rRNA in *Mettl14* KO or *Mettl3* KO cells. We next performed m6A MeRIP on nuclear RNA from *Mettl14* KO and *Mettl3* KO cells. Importantly, for MSR transcripts, we observed a statistically significant reduction of m6A RNA enrichment in both the *Mettl14* KO and a 5-fold reduction in the *Mettl3* KO samples when compared to their WT controls (Figure 3C, left). For LINE L1MdA 5′UTR transcripts, no significant decrease in m6A RNA enrichment was detected in *Mettl14* KO, however a 10-fold reduction was observed in *Mettl3* KO samples (Figure 3C, right). This may suggest that the catalytically inactive Mettl14 could have a more restricted RNA substrate recognition profile or that the much higher m6A RNA levels of LINE L1MdA 5′UTR transcripts (as compared to MSR transcripts) could add to increased RNA stability. Despite these differences, the sum of the data indicate the involvement of the Mettl3/Mettl14 complex in m6A RNA methylation of MSR and LINE L1MdA 5′UTR transcripts in mouse ES cells.

### HiSeq RNA sequencing of m6A enriched repeat RNA reveals MSR transcripts as a novel target for Mettl3/Mettl14-mediated modification

m6A RNA modification was mostly studied on protein coding poly(A) transcripts and only recently were there reports identifying m6A on some repeat RNA (13,75) and on transcripts form endogenous retroviruses (51,52). We asked whether there is a difference in m6A RNA methylation between protein coding and non-coding transcripts and among distinct repeat classes. We performed HiSeq RNA sequencing on m6A-containing transcripts that were enriched by MeRIP of nuclear RNA from WT and *Mettl14* and *Mettl3* KO cells (see Materials and methods). We could confirm that the highest percentage of called m6A peaks were present within the 3′UTR of protein coding poly(A) transcripts in both WT26 (34.1% of all peaks) and RBC (40.7% of all peaks) cells (Supplementary Figure S6A). Motif analysis of the m6A peaks within the 3′UTR identified the previously described RRACH motif (34,35). While the percentage of m6A peaks in 3′UTR of protein-coding transcripts was reduced to 24.7% in *Mettl3* KO ES cells, there was no major change for overall m6A peak calling in the 3′UTR of protein-coding transcripts from *Mettl14* KO cells (Supplementary Figure S6B). However, the intensity of these called m6A 3′UTR peaks was also reduced in the *Mettl14* KO RNA data sets (Supplementary Figure S7).

**Figure 4.**
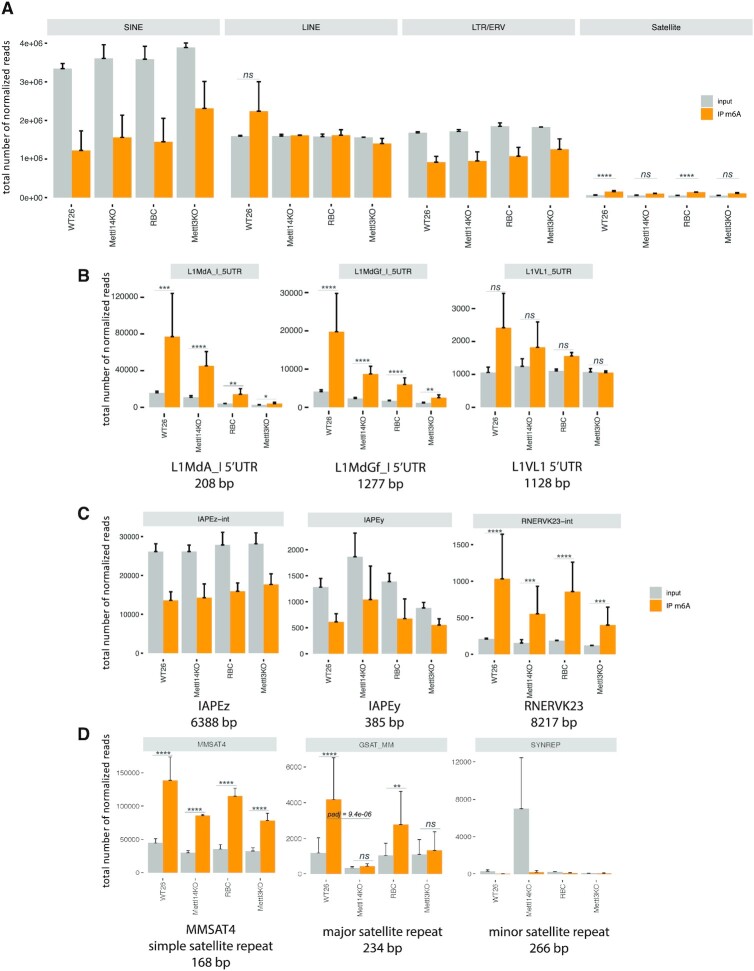
HiSeq RNA sequencing of m6A enriched RNA reveals MSR transcripts as a novel target for Mettl3/14 mediated modification. HiSeq RNA sequencing (75 bp, paired end) was performed on m6A-enriched nuclear RNA from WT26, *Mettl14* KO, RBC and *Mettl3* KO ES cells. Repeat reads were quantified by TEtranscripts algorithms (see Materials and methods) and were classified into the four main repeat classes (SINE repeats, LINE repeats, LTR/ERV retrotransposons and satellite repeats. (**A**) Bioinformatic meta-analysis showing the total number of normalized reads for each repeat class in WT26, *Mettl14* KO, RBC and *Mettl3* KO ES cells. Normalized reads are presented as input (grey bar) and m6A-IP (orange bar). Higher number of normalized reads in the m6A-IP sample over the input sample indicates m6A RNA enrichment. Statistical significance for m6A RNA enrichment (orange bar) over input (grey bar) was determined by the Wald test and *P*-values were corrected for multiple testing with the Benjamini and Hochberg method in DESeq2. Log_2_FC >1 and *P*-values ≤0.05 are considered statistically significant and are indicated by asterisks (*P ≤ 0.05; **P ≤ 0.01; ***P ≤ 0.001; ****P ≤ 0.0001). (**B**) Bioinformatic analysis of m6A RNA enrichment for individual repeat subtypes (LINE L1MdA 5′UTR, LINE L1 MdGf 5′UTR and L1Vl1 5′UTR) within the LINE repeat class. (**C**) Bioinformatic analysis of m6A RNA enrichment for individual repeat subtypes (IAPEz-int, IAPEy and RNERVK23-int) within the LTR/ERV repeat class. (**D**) Bioinformatic analysis of m6A RNA enrichment for individual repeat subtypes (MMSAT4, major satellite repeat and minor satellite repeat) within the satellite repeat class. For panels B, C and D, statistical significance between WT26/*Mettl14* KO and RBC/*Mettl3* KO samples was determined. While most of the differences were not within a statistically significant range (adjusted *P*-values not indicated), the WT26/*Mettl14* KO comparison of MSR transcripts (middle of panel D) is highly significant with an adjusted *P*-value of 9.4e–06. The HiSeq RNA data presented in this Figure were only done for *n* = 2 biological replicates and show the mean ± SD.

We then analyzed m6A peak calling of transcripts that fall within the main repeat classes. In the mouse transcriptome, short interspersed nuclear elements (SINE) are the most abundant class of repeat transcripts (≥ 3 million reads), followed by LINE transcripts, long terminal repeats (LTR)/endogenous retroviruses (ERV) (both > 1 million reads) and satellite repeat transcripts (Figure 4A). m6A enrichment was not detected for SINE and LTR/ERV transcripts, and for LINE transcripts was only apparent in WT26 sample. Although least abundant (<50 000 reads), satellite RNA displays consistent m6A enrichment over input in both the WT26 and RBC samples (Figure 4A, right panel). As this meta-analysis may mask possible changes in m6A RNA enrichment for individual repeat subtypes in each of the main repeat classes, we also examined specific examples for LINE, IAP and LTR/ERV and satellite repeats.

LINE L1MdA 5′UTR (≈75 000 reads in WT26) and L1MdGf 5′UTR (≈20,000 reads in WT26) repeat transcripts showed m6A enrichment over input, and this m6A enrichment was modestly decreased in *Mettl14* KO or reduced by >50% in *Mettl3* KO RNA samples (Figure 4B). By contrast, LINE L1VL1 5′UTR (around 2000 reads in WT26) RNA has only a statistically non-significant m6A enrichment over input (Figure 4B). For the IAP and ERV/LTR repeat class, we did not observe m6A enrichment over input for IAPEz-int (intact) and IAPEy (solitary LTR) transcripts, although there are 12 000 IAPEz reads and 500 IAPEy reads in RNA samples from WT26 cells (Figure 4C). In total RNA preparations IAPEz and IAPEy LTR RNA transcripts were recently shown to be m6A methylated by the Mettl3/Mettl14 complex (51,52). Another ERV, RNERVK23-int (1,000 reads in WT26) displays m6A RNA enrichment over input with an observed 2-fold reduction in *Mettl14*- or *Mettl3*-KO RNA samples (Figure 4C). For satellite repeats, the simple satellite MMSAT4 shows >140 000 RNA reads in WT26 and this m6A enrichment over input is reduced in RNA samples from *Mettl14* and *Mettl3* KO cells (Figure 4D). Major satellite repeat transcripts (annotated as GSAT_MM) are ≈ 4-fold enriched over input and display around 4000 m6A-positive reads in WT26. The m6A enrichment for major satellite repeat transcripts is significantly decreased in *Mettl14* (log_2_ fold change >1, adjusted *P*-value = 9.4e–06) and reduced by half in *Mettl3* KO samples (Figure 4D). Notably, A/T-rich minor satellite repeat transcripts (SYNREP), despite their high A content (>43% A-rich) and vast derepression in the *Mettl14* KO A10 clone, are not enriched for m6A RNA methylation (Figure 4D).

Although we observed significant differences for m6A RNA enrichment between the four main repeat classes and considerable variability among distinct repeat subtypes, the data suggest that some nuclear LINE 5′UTR, ERV-type, and major satellite repeat transcripts are enriched for m6A RNA methylation. In addition, m6A RNA methylation of major satellite repeat transcripts is dependent on Mettl14 and/or Mettl3 function.

### Impaired chromatin association of MSR transcripts in *Mettl14* and *Mettl3* KO ES cells

Previous reports have shown that MSR transcripts are structural components of mouse heterochromatin (8) and facilitate recruitment of heterochromatin proteins, such as HP1α (9) or Suv39h2 (7). To examine if reduced m6A RNA modification of MSR transcripts will impair heterochromatin integrity, we analyzed DAPI-dense foci, H3K9me3 and HP1α by immunofluorescence in *Mettl14* KO and *Mettl3* KO ES cells (Supplementary Figure S8A). We did not observe apparent changes when compared to WT26 and RBC cells. We also performed a MSR-specific H3K9me3 ChIP, which did not reveal differences in H3K9me3 accumulation (Supplementary Figure S8B).

**Figure 5.**
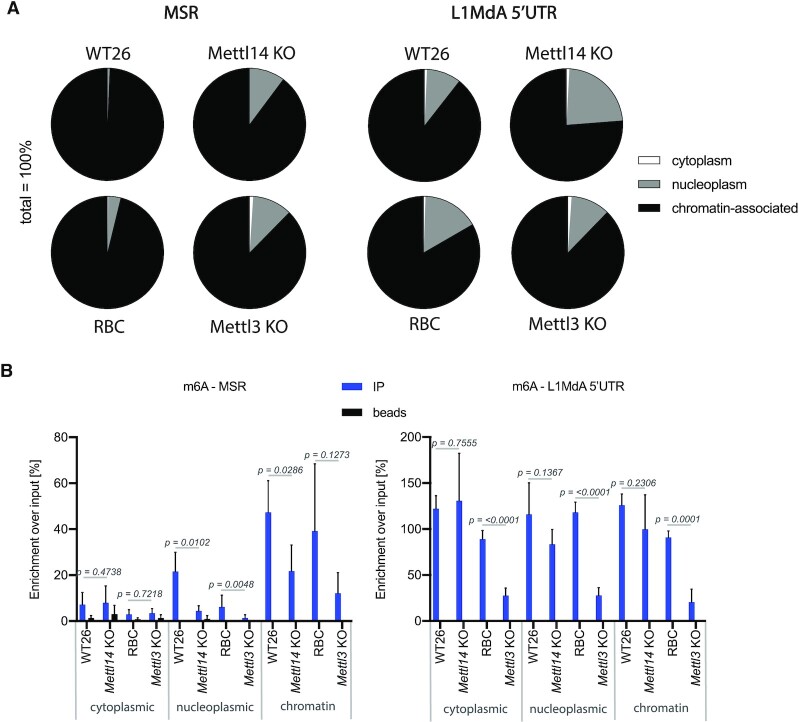
Impaired chromatin association of MSR transcripts in *Mettl14* and *Mettl3* mutant ES cells. (**A**) Pie charts showing the distribution of MSR and LINE L1MdA 5′UTR transcripts in cytoplasmic (white), nucleoplasmic (grey) and chromatin (black) fractions of WT26, *Mettl14* KO, RBC and *Mettl3* KO ES cells. RNA was isolated from each of the subcellular fractions and MSR and LINE L1MdA 5′UTR transcripts were quantified by RT-qPCR. The relative abundance of transcripts in each subcellular fraction is displayed as the percentage of the sum of transcripts in all three subcellular fractions (100%). The data represent the mean from *n* = 3 biological replicates. (**B**) Histogram showing the m6A MeRIP enrichment for m6A-positive MSR (left) and LINE L1MdA 5′UTR (right) transcripts in RNA from cytoplasmic, nucleoplasmic and chromatin fractions of WT26, *Mettl14* KO, RBC and *Mettl3* KO ES cells. The data represent the mean ± SD from *n* = 4 biological replicates and are normalized to the input of each fraction. Statistical significance was determined by unpaired two-tailed t-test and the *P*-values are indicated.

Another important characteristic of MSR transcripts, which allows for the formation of a RNA-nucleosome scaffold and heterochromatic retention of the Suv39h2 KMT, is that they are mostly chromatin-associated (7). To test whether depletion of Mettl14 or Mettl3 would alter the association of hetRNA to chromatin, we isolated RNA from cytoplasmic, nucleoplasmic, and chromatin fractions, followed by RT-qPCR that probes for MSR and LINE L1MdA 5′UTR. Whereas nearly all MSR repeat transcripts are detected in the chromatin fraction of WT26 and RBC cells, their chromatin association is decreased in *Mett14* KO and *Mettl3* KO cells, such that around 10% of MSR RNA is now found in the nucleoplasmic fraction (Figure 5A, left). LINE L1MdA 5′UTR RNA has a subpopulation (10–20%) of transcripts in the nucleoplasm of WT26 and RBC cells and this distribution between nucleoplasm and chromatin did not considerably change in *Mett14* KO and *Mettl3* KO cells (Figure 5A, right). In addition to the illustration with pie charts, we also quantified the data in histograms and controlled the subcellular fractionation by including transcript analysis for *Gapdh* (cytoplasmic and nucleoplasmic) and *Hprt* (mostly nucleoplasmic) (Supplementary Figure S9A). Chromatin-associated MSR transcripts are modestly reduced in both *Mettl14* and *Mettl3* samples as compared to WT26 and RBC and there is a concurrent and statistically significant increase for nucleoplasmic MSR transcripts (Supplementary Figure S9B). This increase in nucleoplasmic RNA is not observed for LINE L1MdA 5′UTR or *Gapdh* or *Hprt* transcripts.

We next performed m6A MeRIP on RNA isolated from the three subcellular fractions. We find that m6A-positive MSR RNA is primarily detected in the chromatin fraction from WT26/RBC samples. The m6A MSR RNA signals in the chromatin fraction are significantly reduced in *Mettl14* KO and also decreased in *Mettl3* KO samples (Figure 5B, left). In addition, there is a gradual decline of m6A-positive MSR transcripts in the nucleoplasmic and cytoplasmic fractions. By contrast, m6A LINE L1MdA 5′UTR RNA is detected to similar levels in all three subcellular fractions. Although there are significantly less m6A-positive LINE L1MdA 5′UTR transcripts in the *Mettl3* KO subcellular fractions, this is not apparent in the *Mettl14* KO samples (Figure 5B, right). These data suggest that m6A RNA methylation status is not discriminating the distribution of m6A-positive LINE L1MdA 5′UTR transcripts within chromatin, nucleoplasm or cytoplasm.

Together, these results indicate that chromatin association of MSR RNA is modestly reduced in the *Mettl14* KO and *Mettl3* KO ES cells. In addition, m6A RNA methylation appears to impart a selective function to MSR transcripts in stabilizing their chromatin association.

### Reduced RNA:DNA hybrid formation of MSR transcripts in *Mettl14* and *Mettl3* mutant ES cells

**Figure 6.**
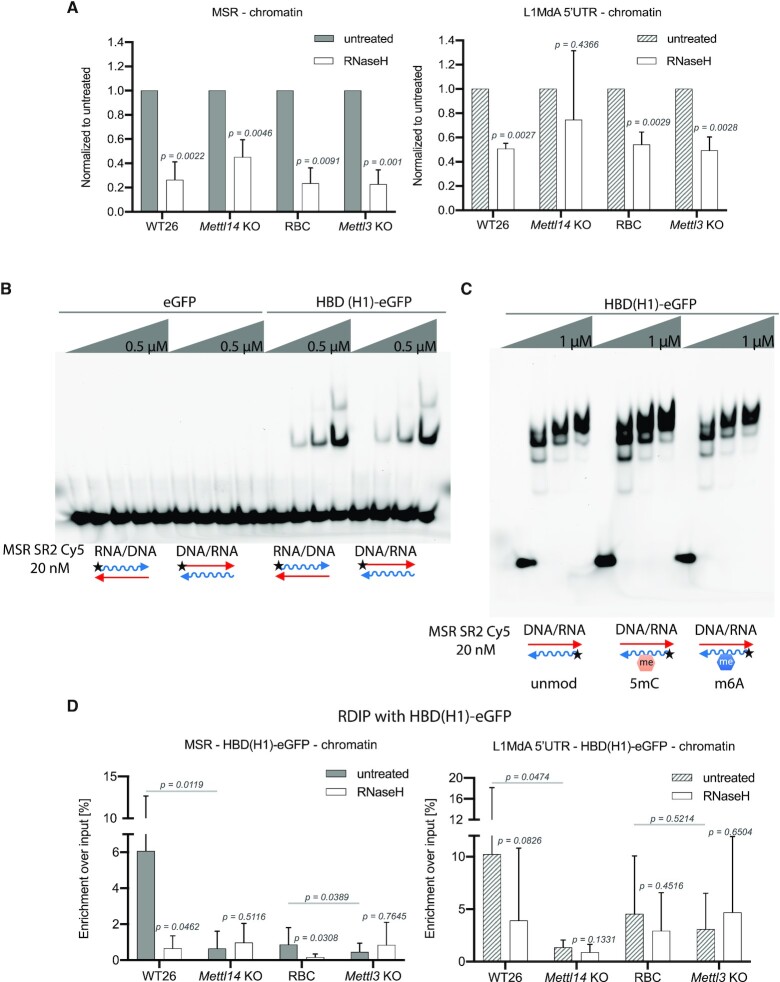
Reduced RNA:DNA hybrid formation of MSR transcripts in *Mettl14* and *Mettl3* mutant ES cells. (**A**) Histogram displaying RNase H sensitivity of chromatin-associated, phenol/chloroform extracted nucleic acids (NA) from WT26, *Mettl14* KO, RBC and *Mettl3* KO ES cells. NA were either untreated (grey or hatched bars) or incubated for 2 h at 37°C with RNase H (NEB) (white bars) and analyzed by RT-qPCR to detect MSR (left) and LINE L1MdA 5′UTR (right) RNA:DNA hybrids. The data represent the mean ± SD from *n* = 4 biological replicates and are normalized to the untreated input in each sample. Statistical significance between untreated and RNase H treated samples was determined by paired two-tailed t-test and the *P*-values are indicated. There are no statistically significant changes for RNase H treated samples between WT26 and *Mettl14* KO or RBC and *Mettl3* KO. (**B**) Electrophoretic mobility shift assay (EMSA) with recombinant eGFP or HBD(H1)-eGFP and RNA:DNA hybrids that were generated by annealing ssRNA (35 nt) or ssDNA oligonucleotides (35 nt) from MSR subrepeat 2. (**C**) EMSA as in (B) with HBD(H1)-eGFP and RNA:DNA hybrids that contain unmodified (unmod) or 5mC modified (5mC) or m6A modified (m6A) RNA oligonucleotides. (**D**) Histogram showing RDIP (using HBD(H1)-eGFP) of chromatin-associated, phenol/chloroform extracted NA from WT26, *Mettl14* KO, RBC and *Mettl3* KO ES cells. Prior to the enrichment with HBD(H1)-eGFP, NA were either untreated (grey or hatched bars) or incubated for 2 h at 4°C with RNase H (Roche) (white bars). MSR (left) and LINE L1MdA 5′UTR (right) RNA:DNA hybrids were detected by RNA amplification using RT-qPCR. The data are calculated as percentage of HBD(H1)-eGFP enriched signal compared to the input signal in each sample. The data represent the mean ± SD from *n* = 5 biological replicates. Statistical significance between untreated and RNase H treated samples was determined by paired two-tailed t-test and the *P*-values are indicated. Statistical significance between untreated WT and untreated mutant samples was determined by unpaired two tailed t-test and the p-values are indicated.

A high proportion of MSR transcripts form RNA:DNA hybrids which are RNase H-sensitive (7). We therefore examined whether m6A RNA methylation could contribute to RNA:DNA hybrid formation of MSR or LINE L1MdA 5′UTR transcripts. Nucleic acids from the chromatin-associated fraction of WT26/RBC and of *Mettl14*/*Mettl3* KO ES cells were either left untreated or treated with RNase H at the same temperature (37°C), and were probed for MSR and LINE L1MdA 5′UTR RNA in RT-qPCR (Figure 6A). RNase H digestion reduced the signal for MSR RNA in all samples (Figure 6A, left). This stringent RNase H sensitivity was attenuated for LINE L1MdA 5′UTR transcripts and not apparent in the *Mettl14* KO samples (Figure 6A, right).

To directly examine RNA:DNA hybrids, we extended the analysis to RNA:DNA hybrid immunoprecipitation (RDIP). There are several described methods to detect RNA:DNA hybrids, but none of them are error-free (22). Thus, we explored a recombinant hybrid-binding domain (HBD) from mouse RNase H1 that we can obtain with high purity (Supplementary Figure S10B). The HBD (RNase H1) was expressed as a HBD(H1)-eGFP-tagged protein that specifically binds to RNA:DNA hybrids, while an eGFP control did not cause any shift of nucleic acids (Figure 6B, left). We also did not observe any binding of HBD(H1)-eGFP to dsDNA or dsRNA (data not shown). Importantly, EMSA performed on RNA:DNA hybrids that contain unmodified or 5mC- or m6A-methylated MSR RNA oligonucleotides displayed comparable binding, indicating that these RNA modifications on RNA:DNA hybrids do not alter the affinity of HBD(H1)-eGFP (Figure 6C).

We performed an additional quality control with chromatin-associated nucleic acids extracted from *p53^−^^/^^−^* and *p53^−^^/^^−^;Rnaseh2^−^^/^^−^* MEFs (70). RDIP with the HBD(H1)-eGFP gave an around three-fold increased signal with chromatin-associated nucleic acids from *p53^−^^/^^−^;Rnaseh2^−^^/^^−^* MEFs as compared to the *p53^−^^/^^−^* control MEFs and this signal was nearly eliminated upon RNase H treatment (Supplementary Figure S10C). This result indicates that the HBD(H1)-eGFP is a high-quality reagent to study RNA:DNA hybrids.

We then used RDIP with HBD(H1)-eGFP of chromatin-associated nucleic acids that were prepared from WT26/RBC and *Mettl14*/*Mettl3* KO ES cells. The chromatin-associated nucleic acids were probed either without or with RNase H treatment prior to the incubation with HBD(H1)-eGFP. For RNase H digestion, we used a protocol with an RNase H enzyme that allows processing of samples at 4°C, as detection of RNA:DNA hybrids by the recombinant HBD(H1)-eGFP is enhanced at 4°C (see Materials and methods). Upon enrichment with HBD(H1)-eGFP, we quantified MSR and LINE L1MdA 5′UTR RNA:DNA hybrids by RNA amplification using RT-qPCR (see also Supplementary Figure S11). MSR RNA:DNA hybrids were detected at high enrichment in chromatin-associated nucleic acids prepared from WT26 cells and, albeit to a much lesser degree, in RBC cells, and these signals were reduced in *Mettl14* KO and *Mettl3* KO samples (Figure 6D, left). RNase H treatment resulted in a decrease of RDIP signal in WT26 and RBC samples, but did not further reduce the RDIP signal in *Mettl14* KO and *Mettl3* KO samples. For LINE L1MdA 5′UTR RNA:DNA hybrids, we observed a comparable RDIP profile, although the RNase H sensitivity was less pronounced as compared to MSR RNA:DNA hybrids. In addition, the differences of LINE L1MdA 5′UTR RDIP signals are statistically significant only for the WT26/*Mettl14* KO comparison (Figure 6D, right).

We also performed RDIP with the S9.6 antibody (76) that is broadly used for the detection of RNA:DNA hybrids. The S9.6 antibody also generated high to intermediate enrichments for MSR RNA:DNA hybrids in samples from the WT26 and RBC samples that are reduced in the *Mettl14* KO but outside a significant difference in the *Mettl3* KO samples (Supplementary Figure S12B, left). With the exception of chromatin-associated nucleic acids prepared from the *Mettl14* and *Mettl3* KO cells, RNase H treatment reduced the RDIP signal in WT26 and RBC samples, although the S9.6-enriched RNA:DNA hybrids in RBC samples appear less sensitive to RNase H digestion as compared to RNA:DNA hybrids that are enriched by the HBD(H1)-eGFP (see Figure 6D, left). The S9.6 antibody is known to bind, in addition to RNA:DNA hybrids, to dsRNA (77) or even triple-helical structures (7). The detection of LINE L1MdA 5′UTR RNA:DNA hybrids with the S9.6 antibody gave only weak (below 0.5% enrichment over input) RDIP signals and there were no detectable differences between WT26/RBC and *Mettl14*/*Mettl3* mutant samples (Supplementary Figure S12B, right).

Despite this variability in the enrichment and RNAse H sensitivity of RNA:DNA hybrids by using either HBD(H1)-eGFP or the S9.6 antibody, our analysis confirms that a significant fraction of chromatin-associated MSR transcripts forms RNA:DNA hybrids. MSR RNA:DNA hybrid formation is reduced in chromatin-associated nucleic acids prepared from *Mettl14* and *Mettl3* KO ES cells, as shown in the RDIP analysis with the HBD(H1)-eGFP and from *Mettl14* KO ES cells as shown in the RDIP analysis with the S9.6 antibody. Together, these results indicate that m6A RNA modification can stabilize MSR RNA:DNA hybrids.

## DISCUSSION

m6A RNA modification has mainly been studied for its function in gene coding transcripts (34,35,39). Only a few studies have begun to analyze the role of m6A methylation in non-coding RNA (50,78–81) or in repeat RNA (13,51,52). No analysis of m6A RNA methylation of MSR repeat transcripts and on its possible role in contributing to heterochromatin stability has been described.

### MSR repeat transcripts are a novel target for m6A RNA methylation

We demonstrate that m6A is an abundant RNA modification of MSR (>20% of transcripts) and LINE L1MdA 5′UTR (>70% of transcripts) repeat RNA (Figure 1). Through a combination of *in vitro* RNA methyltransferase assays (Figure 2) and by comparing the levels of m6A-positive transcripts from WT and mutant ES cells (Figure 3), we show that the Mettl3/Mettl14 complex can target MSR repeat RNA (Figures 2 and 3C). In addition, the unbiased MeRIP-seq analysis for m6A detection in transcripts from the main four repeat classes, confirmed that MSR repeat RNA and some LINE transcript subtypes (e.g. LINE L1MdA 5′UTR) are most consistently enriched (Figure 4). This m6A MeRIP enrichment is attenuated in total RNA preparations from *Mettl14* and *Mettl3* KO ES cells. Moreover, MSR and LINE L1MdA 5′UTR transcripts decline, to variable degrees, in *Mettl14* and *Mettl3* KO ES cells (Figure 3B). This observation is in agreement with a recent study where in ES cell lines that are depleted for components of the Mettl3/Mettl14 complex, the RNA levels for some LINE subtypes decrease, while other repeat transcripts, such as endogenous retroviruses (ERV) are increased (51,52). A reduced level of MSR and LINE L1MdA 5′UTR repeat transcripts in cells with compromised m6A RNA modification suggests a role for m6A in stabilizing these heterochromatic RNA. A stabilizing function for m6A RNA modification was recently reported for an mRNA that is regulated by insulin-like growth factor 2 mRNA-binding proteins 1–3 (IGF2BP1–3) (82). On the other hand, most mRNA, some non-coding transcripts and ERV repeat RNA (primarily IAP retrotransposons) are destabilized by m6A methylation (13,39,51,52). These distinct m6A RNA modification patterns therefore appear to be highly context dependent. Importantly, our study focused on the analysis of RNA modification of repeat transcripts in nuclear RNA and it is possible that Mettl3/Mettl14-dependent m6A RNA methylation is targeted and/or processed differently for nuclear vs. cytoplasmic transcripts. In summary, our data reveal nuclear MSR repeat transcripts as novel targets for m6A RNA methylation and are consistent with a function of m6A to stabilize MSR repeat RNA.

The precise mechanism underlying the specificity of m6A RNA methylation by the Mettl3/Mettl14 complex is still unknown (83). The presence of the RRACH motif does not appear to be the sole prerequisite and we observed less than 50% reduction for m6A RNA methylation with MSR transcripts in which all RRACH motifs were mutated (Figure 2B). In addition, a high A content also seems to be insufficient, since minor satellite repeat transcripts (43% A-rich) are not enriched for m6A modification (Figure 4D), although they are as A/T-rich as MSR transcripts. H3K36me3 decorated chromatin was proposed to guide m6A RNA methylation to some nascent mRNA (84). We have not detected apparent differences in H3K36me3 at the MSR and minor satellite chromatin by ChIP (data not shown). It remains currently unknown whether there may be other histone modifications that could help in directing m6A RNA methylation onto distinct repeat transcripts.

Although a significant fraction of m6A-positive MSR and LINE L1MdA 5′UTR RNA is lost in *Mettl3* KO ES cells, m6A RNA modification is not fully removed from the population of these repeat transcripts (Figures 3C and 4). Therefore, other enzymes are likely to be involved in m6A RNA methylation of hetRNA. For example, METTL16 was shown to methylate adenosines in a distinct hairpin loop structure present in U6 snRNA and in MAT2A mRNA (79), METTL5 was identified as a 18S rRNA methyltransferase (81) and ZCCHC4 methylates the 28S rRNA (80).

### Possible function of m6A MSR repeat transcripts in mouse heterochromatin

The finding that a considerable fraction (i.e. around 20%) of MSR RNA is m6A-modified raised the question to its possible role in the formation and/or stabilization of heterochromatin. Based on the m6A-*Xist* paradigm (50), and as recently described for Mettl3-dependent m6A RNA methylation of IAP retrotransposon repeat transcripts (51,52), m6A-positive MSR RNA could influence binding affinities of factors involved in heterochromatin organization. In addition to Ythdf1/Ythdf2/Ythdf3, there are two nuclear proteins (Ythdc1 and Ythdc2) containing the YTH (YT521-homology) domain (85) that has been shown to directly bind to m6A RNA (86). We probed the recombinant YTH domain of Ythdc1 and Ythdc2 with several m6A modified RNA oligonucleotides but did not detect preferred affinity for m6A MSR RNA (data not shown). We also examined *in vitro* binding of HP1α (8,9) and of the basic domain of Suv39h2 (7) to m6A MSR RNA, however m6A RNA methylation did not change their RNA binding affinities (data not shown). Some hnRNPs bind to partially distorted dsRNA that occur as a result of m6A RNA methylation (78,87). IGF2BP1–3 proteins, fragile X mental retardation protein (FMRP), and proline rich and coiled-coil containing 2a (Prrc2a) were also shown to interact with m6A-modified RNA (85,88,89). Whether these or other factors (90) for m6A RNA binding could provide a heterochromatin-specific interaction with m6A-positive MSR repeat transcripts is currently unknown.

MSR repeat transcripts are chromatin associated and form RNA:DNA hybrids (7). We observed that the chromatin association of MSR RNA is modestly impaired in *Mettl14* KO and *Mettl3* KO ES cells (Figure 5A). Moreover, m6A-positive MSR repeat transcripts appear to be preferably enriched in the chromatin fraction (Figure 5B and Supplementary Figure S9A). This result indicates a selective role for m6A RNA methylation of MSR repeat transcripts in enhancing their chromatin association. In addition, MSR RNA:DNA hybrid formation was also impaired in *Mettl14* and *Mettl3* KO ES cells (Figure 6D and Supplementary Figure S12B). On average, between 50–70% of chromatin-associated MSR repeat transcripts are RNase H sensitive (Figure 6A). Intriguingly, the RNase H sensitivity of HBD(H1)-eGFP-enriched repeat transcripts was more pronounced for MSR RNA:DNA hybrids as compared to LINE L1MdA 5′UTR RNA:DNA hybrids and therefore suggests another function for m6A RNA methylation in stabilizing the RNA within an MSR RNA:DNA hybrid structure. Indeed, m6A RNA methylation was described to promote R-loop formation on mRNA (91) and a recent study showed METTL3-dependent m6A RNA methylation to increase the accumulation of RNA:DNA hybrids at DNA double strand breaks (92). m6A RNA methylation favors the conversion of paired to unpaired RNA and relaxes the secondary structure of the dsRNA (78,93). MSR repeat transcripts have a distinct secondary structure with extended single-stranded regions which has been proposed to facilitate chromatin association via RNA:DNA hybrid formation (7). Our *in vitro* assays indicate that m6A-modified MSR RNA oligonucleotides have a modestly increased potential (as compared to unmodified MSR RNA oligonucleotides) to form RNA:DNA hybrids or to invade a double-stranded DNA target (Supplementary Figure S13). Thus, the additional stabilization of an unpaired MSR RNA structure by m6A RNA methylation would further augment the potential to form RNA:DNA hybrids and strengthen the function of MSR repeat RNA at mouse heterochromatin.

Several studies have implicated phase separation as one of the mechanisms to modulate heterochromatin organization (94,95), and m6A modified RNA was recently shown to enhance the ability of some RNA/protein complexes to phase separate (96). Although other models for dynamic transitions between distinct heterochromatic states have also been reported (97), we have not explored, if, or to what extent, m6A RNA methylation of MSR repeat transcripts could contribute to a more relaxed or compact heterochromatin structure. We have analyzed HP1α localization and H3K9me3 accumulation, but did not find apparent changes between WT26/RBC and *Mettl14* and *Mettl3* KO ES cells (Supplementary Figure S8). Mammalian heterochromatin is a very robust sub-nuclear compartment that is organized and protected by several independent mechanisms including DNA methylation, histone modifications, histone variant turnover, chromatin-associated non-coding RNA and its higher-order structuring into focal domains (2,98). The impairment of any one of these regulatory pathways is probably not sufficient to destabilize heterochromatin. Additional studies on a full depletion of m6A RNA methylation of MSR repeat transcripts are required to further dissect the functions of m6A MSR RNA within these other regulatory mechanisms that together maintain the integrity of mammalian heterochromatin.

## DATA AVAILABILITY

The MeRIP-seq and the RNA-seq data have been deposited to the GEO repository (https://www.ncbi.nlm.nih.gov/geo/query/acc.cgi), with the accession number GSE156481.

## Supplementary Material

gkab364_Supplemental_File

## Notes

Present address: Valentina Perrera, Scuola Internazionale Superiore di Studi Avanzati (SSIA), Trieste 34136, Italy.

Present address: Megumi Onishi-Seebacher, Novartis Institute for Biomedical Research (NIBR), Basel 4056, Switzerland.

Present address: Katharina Fritz, BioNTech RNA Pharmaceuticals GmbH, Mainz 55131, Germany.

Present address: Philip Knuckles, Scailyte AG, Sursee 6210, Switzerland.
