# Supplementary material for: m6A RNA methylation of major satellite repeat transcripts facilitates chromatin association and RNA:DNA hybrid formation in mouse heterochromatin
Source: Nucleic Acids Res. 2021 May 17;49(10):5568–87. doi: 10.1093/nar/gkab364 (PMC8191757; doi:10.1093/nar/gkab364)
Supplement: gkab364_Supplemental_File [file gkab364_supplemental_file.pdf]

SUPPLEMENTARY DATA

SUPPLEMENTARY MATERIALS AND METHODS

MiSeq RNA sequencing of captured hetRNA

6 ng of MSR-captured RNA and 3 ng of LINE L1MdA 5'UTR-captured RNA were fragmented and then used to generate cDNA libraries (ribosomal RNA-depleted) for MiSeq analysis with the NEBNext Ultra Directional RNA Library Prep Kit for Illumina (NEB). Because of their different size distribution, the MSR-captured RNA was divided into two tubes, sheared in NEBNext First Strand Synthesis Reaction buffer (NEB) for 7.5 min and 8.5 min and then combined again. The LINE L1MdA 5'UTR-captured RNA was fragmented for 8.5 min. The cDNA libraries were sequenced on a MiSeq (Illumina) platform using a 75 bp paired end multiplex run with a coverage of around 3.5 million reads per cDNA library.

Indirect immunofluorescence for HP1α and H3K9me3

$5x10^4$ ES cells were spun on a microscope slide using a Cytospin 4 (Thermo Fisher Scientific). Cells were fixed with 2% paraformaldehyde (PFA) for 10 min at RT, washed three times with PBS and then stored at 4°C. Cells were permeabilized with 0.5% Triton X-100 (in PBS) for 5 min at RT and washed three times with PBS. The HP1α antibody (mouse monoclonal, Merck Millipore, clone 15.19s2) was diluted 1:200 in 5% goat serum and the H3K9me3 antibody (rabbit polyclonal, Abcam, cat. no. ab8898) was diluted 1:500 in 5% goat serum. The primary antibodies were incubated with the permeabilized cells for 1 h at RT or O/N at 4°C. After three washes with PBS, Cy3-coupled goat anti-mouse polyclonal antibody and Cy5-coupled goat anti-rabbit polyclonal antibody (both Thermo Fisher Scientific) were added in a 1:250 dilution in 5% goat serum and incubated for 1 h at RT in a dark chamber. The secondary antibodies were washed three times with PBS. Cells were post-fixed with 2% paraformaldehyde (PFA) for 10 min in RT and mounted with Vectashield medium containing DAPI (Vector Laboratories). The coverslips were sealed with nail polish and stored at 4°C. Immunofluorescence was analyzed with the Observer Z.1 fluorescence microscope (Zeiss) using a 63x magnification and the AxioCam MRm camera (Zeiss). Images were acquired and analyzed with Axio Imager software version 2 (Zeiss).

Chromatin immunoprecipitation (ChIP)

ChIP was performed as described (1) with the following adjustments. 1-2 maxi dishes with $1x10^7$ ES cells were agitated in cell culture crosslinking solution containing a final concentration of 1% formaldehyde (Sigma-Aldrich) for 10 min at RT. Crosslinking was quenched with 0.125 M glycine for 5 min and the cells were washed 3 times with ice-cold PBS, and then lysed with 1 ml of ChIP-lysis buffer (1% SDS, 10 mM EDTA, 50 mM Tris-HCl pH 8.0, and protease inhibitors (Roche)). Cell lysates were transferred to miliTUBEs (Covaris) and sonicated using a Covaris S220 with the following settings: 105 Peak Power, 5.0 Duty Factor, 200 cycles per burst, and 240 s sonication time. DNA from an aliquot of the sample was run on a 1% agarose gel to control for the generation of sonicated DNA fragments

ranging between 200-1000 bp. The sonicated chromatin was then stored at 4°C. Sonicated chromatin corresponding to 3 µg of DNA was incubated in 1 ml of dilution buffer (1% Triton X-100, 2mM EDTA pH 8.0, 150 mM NaCl, 20 mM Tris-HCl pH 8.0, and protease inhibitors (Roche)) with 25 µl of magnetic Protein G Dynabeads (Thermo Fisher Scientific) that had been coupled to 3 µg of α-H3K9me3 antibody (Abcam, cat. no. ab8898) O/N at 4°C. The next day, the bead-antibody-chromatin complexes were washed three times for 5 min at RT with 1 ml of ChIP-wash buffer (0.1% SDS, 1% Triton X-100, 2mM EDTA pH 8.0, 150 mM NaCl, 20 mM Tris-HCl pH 8.0, and protease inhibitors (Roche)) and once for 10 min with 1 ml of ChIP-final wash buffer (0.1% SDS, 1% Triton X-100, 2mM EDTA pH 8.0, 500 mM NaCl, 20 mM Tris-HCl pH 8.0, and protease inhibitors (Roche)). Beads were resuspended in 100 µl of ChIP-elution buffer (1% SDS and 0.1M $NaHCO_3$) containing 0.5 µg/µl RNase A and 0.5 µg/µl Proteinase K (both Thermo Fisher Scientific) and incubated for 1 h at 37°C, followed by a 5 h or O/N incubation at 65°C to reverse the crosslinking. DNA was then purified with a NucleoSpin Gel and PCR Clean Up DNA purification kit (Macherey-Nagel) and eluted in 30 µl water. 2 µl were processed for qPCR with the QuantStudio 6 Flex machine (Applied Biosystems) with an annealing temperature of 60°C in a program using 40 cycles.

Expression analysis of protein-coding genes in nuclear RNA

Nuclear RNA of WT26, *Mettl14* KO, RBC and *Mettl3* KO ES cells was isolated and processed for HiSeq RNA sequencing as described in Materials and methods. The quality of raw and trimmed Fastq reads was verified using FastQC (2). Reads were trimmed using cutadapt version 1.8.1 (3). Reads were then aligned to the GRCm38 genome from Ensembl using STAR version 2.6.0c (4). Differential analysis was carried out using DESeq2 version 1.26.0 (5) on the count matrices output from snakePipes version 2.1.0 (6). For MA plots, transcripts with a baseMean > 50 reads were considered and significant differences in gene expression have absolute(log2FC) > 1 changes and adjusted p-values of < 0.05. The p-values attained by the Wald test are corrected for multiple testing using the Benjamini and Hochberg (BH) method. Data visualization was performed using ggplot2 (7). Heatmaps for gene expression of genes encoding core components of chromatin, transcription machinery and m6A RNA pathway were generated using pheatmap in R (pheatmap: Pretty heatmaps. R package version 1.0.8. https://CRAN.Rproject.org/package=pheatmap). GO enrichment analysis was performed with the enrichGO function from clusterProfiler R package version 3.14.3 (8) using the Biological Process ontology. P-values were adjusted using the BH method. Dotplots represent significant pathways (adjusted p-value < 0.05).

Intensity plots for called m6A peaks in the 3'UTR of protein–coding transcripts

Intersection of the called m6A peaks in the 3' UTR of protein-coding transcripts between WT26 and *Mett14* KO (6793 common peaks) and between RBC and *Mett3* KO (2240 common peaks) data sets (see supplementary Figure S7) was performed using bedtools2 intersect version 2.27.0 (9). Coverage of the bam files normalized to the sequencing depth and inputs was computed using deepTools version 3.5.0 bamCompare (10). DeepTools computeMatrix reference point was used to generate scores per genomic region (intersection of the peaks) 3kb upstream and 3kb downstream from the center of the

peak. The heatmaps and profile of those regions were generated using deepTools plotProfile and represent the normalized coverage (intensity).

In vitro RNA:DNA hybrid formation assay

For *in vitro* RNA:DNA hybrid formation, we used a 35 bp sequence from the subrepeat 2/3 of the MSR. This sequence codes for a RNA oligonucleotide in the forward orientiation (MSR-SR2/3-FOR) that contains 10 adenosines. The MSR-SR2/3-FOR and a modified RNA oligonucleotide in which all 10 adenosines were replaced by m6A (MSR-SR2/3-FOR(m6A)) were ordered from Sigma-Aldrich, as well as the corresponding DNA forward and DNA reverse oligonucleotides. 5 µM of DNA reverse oligonucleotide was mixed with increasing concentrations (0.1 µM to 10 µM) of either unmodified or m6A-modified RNA forward oligonucleotide in 1x MES buffer (2-(N-morpholino)ethanesulfonic acid 0.5 M, pH 7). Samples were denatured for 3 min at 90°C, annealed for 30 min at 37°C, incubated for 1 h at 4°C and then stored at -20°C. RNA:DNA hybrids were resolved on a 10% native polyacrylamide (19:1) gel (70 mM Tris-borate, 1.5 mM EDTA, 1% APS, 0.15% TEMED) in 0.5x TBE buffer (45 mM Tris-borate, 1 mM EDTA), stained for 15 min with 0.5x of SYBR Gold Nucleic Acid Gel Stain (Thermo Fisher Scientific) in 0.5x TBE buffer and visualized under UV light. Nucleic acids were blotted to a Hybond N+ nylon membrane (GE Healthcare) and incubated with the S9.6 antibody (Kerafast) at a 1:1,000 dilution. A goat α-mouse HRP-conjugated secondary antibody (Jackson Immuno Research) was used at a 1:2,500 dilution and the signal was detected using Amersham ECL Western Blotting Detection Reagent (GE Healthcare). For the invasion of ssRNA to a dsDNA template, similar conditions were used, with the difference that a constant concentration (5 µM) of annealed dsDNA was titrated with increasing concentrations (0.5 µM to 10 µM) of either unmodified or m6A-modified RNA forward oligonucleotide. The sequences of the RNA and DNA oligonucleotides used for the assay are listed in Table 1.

Table 1. Oligonucleotides used in the study

Name	Sequence 5'->3'	Method
MSR one repeat FOR	TGGAATATGGCGAGAAAACTG	RT-qPCR, qPCR
MSR one repeat REV	AGGTCCTTCAGTGGGCATTT	RT-qPCR, qPCR
L1MdA 5'UTR FOR	ACTGCGGTACATAGGGAAGC	RT-qPCR, qPCR
L1MdA 5'UTR REV	TGTGATCCACTCACCAGAGG	RT-qPCR, qPCR
EGFP IVT FOR	GGCAACTACAAGACCCGCGCC	RT-qPCR
EGFP IVT REV	GCCCTTCAGCTCGATGCGGTT	RT-qPCR
Klf4 FOR	TTTGTAAGTCCGGGCATGTT	RT-qPCR
Klf4 REV	GGGGTCTGATACTGGATGGA	RT-qPCR
Nanog FOR	GGACTTTCTGCAGCCTTACG	RT-qPCR
Nanog REV	GCTTCCAAATTCACCTCCAA	RT-qPCR
Sox2 FOR	ACATGTGAGGGCTGGACTG	RT-qPCR
Sox2 REV	TTTTGCACCCCTCCCAATTC	RT-qPCR
Pou5f1 FOR	GTGGAGGAAGCCGACAACAATGA	RT-qPCR
Pou5f1 REV	CAAGCTGATTGGCGATGTGAG	RT-qPCR
Gapdh FOR	TGAACGGGAAGCTCACTGG	RT-qPCR
Gapdh REV	TCCACCACCCTGTTGCTGTA	RT-qPCR
28S rRNA FOR	GTAACTATGACTCTCTTAAGGTAGCCA	RT-qPCR
28S rRNA REV	CTTCACCGTGCCAGACTAGAG	RT-qPCR

Hprt FOR	AGTGATAGATCCATTCCTATGACTGTAG	RT-qPCR
Hprt REV	GTTAAAGTTGAGAGATCATCTCCACC	RT-qPCR
Zfp180 ex 5 FOR	CCGTACAGGTGCAATCTGTG	qPCR
Zfp180 ex 5 REV	GTTTGTAGCTCTGGCGGAAC	qPCR
β-Actin prom FOR	TTTTATGGCTCGAGTGGCCG	qPCR
β-Actin prom REV	CTGCAAAGAAGCTGTGCTCG	qPCR
Mettl3 ex 5 gRNA top	CACCGTGGAAAGAGTCGATCAGCAC	CRISPR sgRNA
Mettl3 ex 5 gRNA bottom	AAACGTGCTGATCGACTCTTTCCAC	CRISPR sgRNA
Mettl14 ex 10 gRNA top	CACCGCGTGAAGCGAAGCACAGACG	CRISPR sgRNA
Mettl14 ex 10 gRNA bottom	AAACCGTCTGTGCTTCGCTTCACGC	CRISPR sgRNA
Mettl3 ex5 FOR	GCCAGGAGCTTGCTCTTACAC	CRISPR PCR
Mettl3 ex5 REV	GTGGGTCAGCCATCACAACTG	CRISPR PCR
Mettl14 ex10 FOR	ACGTGGTGTGGGAAATCCTG	CRISPR PCR
Mettl14 ex10 REV	ATCAAAACTGAGGTCTCACCTGG	CRISPR PCR
L1MdA_1	CGATTGGATTGGGGCAGG-biotin	RNA capture for MS
L1MdA_2	AGTCGAGCGGAAGGGACT-biotin	RNA capture for MS
L1MdA_3	ACCAGAGGTCTTAGGGTC-biotin	RNA capture for MS
GSAT_R/AS_1	GAGAAACATCCACTTGACGA-biotin	RNA capture for MS
GSAT_R/AS_3	CTGTAGGACGTGGAATATGG-biotin	RNA capture for MS
GSAT_R/AS_5	GGCGAGGAAAACTGAAAAAGG-biotin	RNA capture for MS
GSAT_R/AS_7	GGCGAGAAAACTGAAAATCACG-biotin	RNA capture for MS
attB1_5'_NLS_ FOR_GW	GGGGACAAGTTTGTACAAAAAAGCAGGCTTCCCG AAGAAAAAGCGTAAAGTG	protein cloning
attB2_3'_eGFP_ REV_GW	GGGGACCACTTTGTACAAGAAAGCTGGGTCTTAC TTGTACAGCTCGTCCATGC	protein cloning
MSR_SR2_DF_ Cy5	(Cy5)GAAATATGGCGAGGAAAACTGAAAAAGGTG GAAAA	EMSA
MSR_SR2_DR	TTTTCCACCTTTTTCAGTTTTCCTCGCCATATTTC	EMSA
MSR_SR2_RF_ Cy5 (RNA)	(Cy5)GAAAUAUGGCGAGGAAAACUGAAAAAGGU GGAAAA	EMSA
MSR_SR2_RR_ Cy5 (RNA)	(Cy5)UUUUCCACCUUUUUCAGUUUUCCUCGCCA UAUUUC	EMSA
MSR_SR2_RR_ Cy5_5mC (RNA)	(Cy5)UUUU[5mC] [5mC]A[5mC] [5mC]UUUUU[5mC]AGUUUU[5mC] [5mC]U[5mC]G[5mC] [5mC]AUAUUU[5mC]	EMSA
MSR_SR2_RR_ Cy5_m6A (RNA)	(Cy5)UUUUCC[m6A]CCUUUUUC[m6A]GUUUUCCU CGCC[m6A]U[m6A]UUUC	EMSA
MSR_SR2/3_RF_ m6A (RNA)	UUU[m6A]G[m6A]U[m6A]UGUCC[m6A]CUGU[m6A] GG[m6A]C[m6A]UGG[m6A][m6A]U[m6A]UGGC	RNA:DNA hybrid formation
MSR_SR2/3_RF (RNA)	UUUAGAUAUGUCCACUGUAGGACAUGGAAUAUG GC	RNA:DNA hybrid formation
MSR_SR2/3_DF	TTTAGATATGTCCACTGTAGGACATGGAATATGG C	RNA:DNA hybrid formation
MSR_SR2/3_DR	GCCATATTCCATGTCCTACAGTGGACATATCTAAA	RNA:DNA hybrid formation

Reagents

Antibodies

α-5mC, clone 10G4 (Zymo Research, USA, discontinued)
α-m6A (Abcam, UK, # ab190886)
S9.6 (Merck Millipore, USA, # MABE 1095)
α-HP1α, clone 15.19s2 (Merck Millipore, USA, # 05-689)
α-H3K9me3 (Abcam, UK, # ab8898)

Kits

RNeasy MinElute Cleanup (Qiagen, Germany, #74204)
SuperScript II Reverse Transcriptase (Thermo Fisher Scientific, USA, #18064022)
Bioanalyzer RNA 6000 Pico (Agilent, USA, #5067-1513)
Bioanalyzer RNA 6000 Nano (Agilent, USA, # 5067-1511)
Quant-iT Ribo-Green RNA Assay (Thermo Fisher Scientific, USA, #R11490)
MEGAScript T7 Transcription Kit (Thermo Fisher Scientific, USA, #AMB13345)
Qubit RNA High Sensitivity Assay (Thermo Fisher Scientific, USA, #Q32852)
TruSeq Stranded Total RNA Library Prep Gold (Illumina, USA, #20020596)
Gateway system (Thermo Fisher Scientific, USA, #12535029 and #11791019)
NEBNext Ultra Directional RNA Library Prep Kit for Illumina (NEB, USA, #E7420S)
NucleoSpin Gel and PCR Clean Up Columns (Macherey-Nagel, Germany, #740609.250)
RNA Clean & Concentrator-5 (Zymo Research, USA, #R1013)

Recombinant proteins

METTL3/METTL4 (Active Motif, USA, #31570)
6xHis-MBP-NLS-HBD(H1)-eGFP (Jenuwein lab)
6xHis-MBP-NLS-eGFP (Jenuwein lab)

Biological resources

Cell lines

WT26 ES cells (Jenuwein lab)
Suv39h dn ES cells (Jenuwein lab)
J1 ES cells (11)
Mettl14 KO (Jenuwein lab)
RBC (12)
Mettl3 KO (12)
p53$^{-/-}$ MEFs (13)
p53$^{-/-}$, *Rnaseh2*$^{-/-}$ (13)
COS-7 cells (ATCC, USA, #CRl-1651) expressing LIF (Jenuwein lab)

Bacterial strains

Rosetta *E.coli* (Merck Millipore, USA, #70954)

Plasmid vectors

pSAT-MSR-1-repeat (14)

pEX-L1MdA-1-repeat (15)

pCR4-TOPO-EGFP-S1 (Jenuwein lab)

pSpCas9(BB)-2A-Puro (pX459, Addgene, USA, #62988) (16)

pDEST-HisMBP (Addgene, USA, #11085) (17)

SUPPLEMENTARY REFERENCES

1. Bulut-Karslioglu, A., De La Rosa-Velazquez, I.A., Ramirez, F., Barenboim, M., Onishi-Seebacher, M., Arand, J., Galan, C., Winter, G.E., Engist, B., Gerle, B. *et al.* (2014) Suv39h-dependent H3K9me3 marks intact retrotransposons and silences LINE elements in mouse embryonic stem cells. *Mol Cell*, **55**, 277-290.
2. Andrews, S. (2010) FastQC: a quality control tool for high throughput sequence data. *http://www.bioinformatics.babraham.ac.uk/projects/fastqc*.
3. Martin, M. (2011) Cutadapt removes adapter sequences from high-throughput sequencing reads. *2011*, **17**, 3.
4. Dobin, A., Davis, C.A., Schlesinger, F., Drenkow, J., Zaleski, C., Jha, S., Batut, P., Chaisson, M. and Gingeras, T.R. (2013) STAR: ultrafast universal RNA-seq aligner. *Bioinformatics (Oxford, England)*, **29**, 15-21.
5. Love, M.I., Huber, W. and Anders, S. (2014) Moderated estimation of fold change and dispersion for RNA-seq data with DESeq2. *Genome biology*, **15**, 550.
6. Bhardwaj, V., Heyne, S., Sikora, K., Rabbani, L., Rauer, M., Kilpert, F., Richter, A.S., Ryan, D.P. and Manke, T. (2019) snakePipes: facilitating flexible, scalable and integrative epigenomic analysis. *Bioinformatics (Oxford, England)*, **35**, 4757-4759.
7. Wickham, H. (2009) *Ggplot2: Elegant Graphics for Data Analysis. 2nd Edition*. Springer, New York.
8. Yu, G., Wang, L.G., Han, Y. and He, Q.Y. (2012) clusterProfiler: an R package for comparing biological themes among gene clusters. *Omics : a journal of integrative biology*, **16**, 284-287.
9. Quinlan, A.R. and Hall, I.M. (2010) BEDTools: a flexible suite of utilities for comparing genomic features. *Bioinformatics (Oxford, England)*, **26**, 841-842.
10. Ramírez, F., Ryan, D.P., Grüning, B., Bhardwaj, V., Kilpert, F., Richter, A.S., Heyne, S., Dündar, F. and Manke, T. (2016) deepTools2: a next generation web server for deep-sequencing data analysis. *Nucleic Acids Res*, **44**, W160-165.
11. Li, E., Bestor, T.H. and Jaenisch, R. (1992) Targeted mutation of the DNA methyltransferase gene results in embryonic lethality. *Cell*, **69**, 915-926.
12. Knuckles, P., Carl, S.H., Musheev, M., Niehrs, C., Wenger, A. and Buhler, M. (2017) RNA fate determination through cotranscriptional adenosine methylation and microprocessor binding. *Nat Struct Mol Biol*, **24**, 561-569.
13. Reijns, M.A., Rabe, B., Rigby, R.E., Mill, P., Astell, K.R., Lettice, L.A., Boyle, S., Leitch, A., Keighren, M., Kilanowski, F. *et al.* (2012) Enzymatic removal of ribonucleotides from DNA is essential for mammalian genome integrity and development. *Cell*, **149**, 1008-1022.
14. Lehnertz, B., Ueda, Y., Derijck, A.A., Braunschweig, U., Perez-Burgos, L., Kubicek, S., Chen, T., Li, E., Jenuwein, T. and Peters, A.H. (2003) Suv39h-mediated histone H3 lysine 9 methylation directs DNA methylation to major satellite repeats at pericentric heterochromatin. *Current biology : CB*, **13**, 1192-1200.
15. Velazquez Camacho, O., Galan, C., Swist-Rosowska, K., Ching, R., Gamalinda, M., Karabiber, F., De La Rosa-Velazquez, I., Engist, B., Koschorz, B., Shukeir, N. *et al.* (2017) Major satellite repeat RNA stabilize heterochromatin retention of Suv39h enzymes by RNA-nucleosome association and RNA:DNA hybrid formation. *Elife*, **6**.

16. Ran, F.A., Hsu, P.D., Wright, J., Agarwala, V., Scott, D.A. and Zhang, F. (2013) Genome engineering using the CRISPR-Cas9 system. *Nature protocols*, **8**, 2281-2308.
17. Nallamsetty, S., Austin, B.P., Penrose, K.J. and Waugh, D.S. (2005) Gateway vectors for the production of combinatorially-tagged His6-MBP fusion proteins in the cytoplasm and periplasm of Escherichia coli. *Protein science : a publication of the Protein Society*, **14**, 2964-2971.
18. Batista, P.J., Molinie, B., Wang, J., Qu, K., Zhang, J., Li, L., Bouley, D.M., Lujan, E., Haddad, B., Daneshvar, K. *et al.* (2014) m(6)A RNA modification controls cell fate transition in mammalian embryonic stem cells. *Cell Stem Cell*, **15**, 707-719.

SUPPLEMENTARY FIGURE LEGENDS

Supplementary Figure S1. Workflow and quality control for MeRIP in the detection of 5mC or m6A containing hetRNA. (A) Workflow for the optimization of the MeRIP protocol to detect 5mC or m6A RNA modification of MSR and LINE L1MdA 5'UTR repeat transcripts in nuclear RNA from WT26 or *Suv39h* dn mouse ES cells. **(B)** m6A MeRIP analysis of control mRNA transcripts that have been shown to be m6A-positive (*Klf4*, *Nanog*, *Sox2*) or m6A-negative (*Pou5f1*) (18). Enrichment is calculated as the percentage of m6A-positive transcripts over the total amount of input transcripts (left panel). As an additional control, 1 ng of fully m6A methylated *EGFP in vitro* transcripts (IVT) were spiked into nuclear RNA of WT26 and *Suv39h* dn ES cells, followed by detection with m6A MeRIP (right panel). The data represent the mean ± SD from n=3 biological replicates.

Supplementary Figure S2. Workflow and quality control for the capture of MSR reverse transcripts and of LINE L1MdA 5'UTR forward transcripts. (A) Workflow for the capture of MSR reverse and LINE L1MdA 5'UTR forward transcripts in nuclear RNA from J1 WT ES cells by biotinylated DNA probes. 6 ng of MSR-captured RNA and 3 ng of LINE L1MdA 5'UTR-captured RNA was used in a MiSeq analysis to control for the quality and specificity of the enriched sequences prior to LC-MS/MS mass spectrometry. **(B)** MiSeq sequencing results for MSR-captured RNA (left) and for LINE L1MdA 5'UTR-captured RNA (right) are displayed as reads per kilobase of transcript, per million mapped reads (RPKM) for the most highly enriched RNA sequences.

Supplementary Figure S3. Generation and confirmation of the CRISPR/Cas9 mediated *Mettl14* mutation in the A10 ES cell clone. (A) Schematic diagram of the *Mettl3* and *Mettl14* gene loci and domain organization of their encoded proteins. The MT-A70 domain is encoded by exons 5-11 for *Mettl3* and by exons 8-11 for *Mettl14*. We used sgRNAs targeting exon 5 of *Mettl3* and exon 10 of *Mettl14*. Primers used for the surveyor assay and for genotyping are depicted by black arrows, as are the lengths (in bp) of their PCR amplicons. **(B)** Surveyor assay on genomic DNA of WT26 ES cells (mixed cell population) co-transfected with *Mettl3* and *Mettl14* sgRNA (+ sgRNA) compared to transfection control (- sgRNA). White asterisks indicate mutated PCR products. **(C)** Sequence analysis of exon 10 of *Mettl14* in PCR products derived from WT26 and A10 clone genomic DNA. For the A10 ES cell clone, only mutated PCR sequences were obtained, indicating a homozygous mutation. This homozygote mutation

is a 4 bp deletion (highlighted in blue) that results in a premature stop codon leading to a truncated (311 aa) Mettl14 protein with an impaired MT-A70 domain.

Supplementary Figure S4. Analysis of gene expression in WT26, *Mettl14* KO, RBC and *Mettl3* KO ES cells. HiSeq RNA sequencing (75 bp, paired end) was performed on nuclear RNA from WT26, *Mettl14* KO, RBC and *Mettl3* KO ES cells. Reads were quantified using TEtranscripts and differentially expressed genes were determined using DESeq2 (see Materials and methods). **(A)** MA-plots depicting changes in genes expression in *Mettl14* KO relative to WT26 (left) and *Mettl3* KO relative to RBC (right) ES cells. The total number of up- and downregulated genes is indicated in the plots. **(B)** Gene ontology (GO) analysis of genes differentially expressed in *Mettl14* KO relative to WT26 (left) and *Mettl3* KO relative to RBC (right) ES cells. GO terms (biological processes) with differentially expressed genes are named on the left side of the plots. The x-axis indicates the percentage of differentially expressed genes in a given GO term. The size of the dot specifies the total number of genes annotated in a given GO term. All data represent the mean from n=2 biological replicates.

Supplementary Figure S5. Heat maps of differential gene expression for genes encoding core components of chromatin, the transcription machinery and the m6A RNA pathway in WT26/*Mettl14* KO and in RBC/*Mettl3* KO ES cells. HiSeq RNA sequencing (75 bp, paired end) was performed on nuclear RNA from WT26, *Mettl14* KO, RBC and *Mettl3* KO ES cells. Reads were quantified using TEtranscripts and differentially expressed genes were determined using DESeq2 (see Materials and methods). A canon of genes encoding described core components of chromatin (around 200 genes), the transcription machinery (around 200 genes) and the m6A RNA pathway (around 30 genes) was selected and heat maps for their differential gene expression between WT26/*Mettl14* KO and between RBC/*Mettl3* KO ES cells were generated. Only genes with a baseMean expression of > 50 reads are shown and of those only very few have a statistically significant (adjusted p-value < 0.05 and absolute(log2FoldChange) > 1) change in gene expression between WT26/*Mettl14* and RBC/*Mettl3* ES cells (marked by an asterisk). There were also no significant alterations for expression of *RNaseH1* and *RNaseH2* genes. The data are form from n=2 biological replicates (rep1 and rep2).

Supplementary Figure S6. Bioinformatic analysis of m6A enriched non-repeat RNA. HiSeq RNA sequencing libraries (75 bp, paired-end) were prepared from m6A-enriched nuclear RNA of WT26/RBC and of *Mettl14*/*Mettl3* KO ES cells. Pie charts indicate the distribution of called m6A peaks in non-repeat RNA from WT26 and RBC **(A)** and from *Mettl14* KO and *Mettl3* KO **(B)** ES cells. The total number of called m6A peaks detected on the sense (+) and antisense (-) RNA strands is indicated above the pie charts. In WT26 and RBC samples, the RRACH motif (boxed) is the most prevalent sequence motif underlying m6A peaks in 3'UTR regions of non-repeat RNA. The data represent the mean from n=2 biological replicates.

Supplementary Figure S7. Intensity plots for m6A peak calling in the 3'UTR of coding transcripts from WT26/*Mettl14* KO and from RBC/*Mettl3* KO samples. The data were derived from Figure S6 and called m6A peaks from the segment for enrichment in 3'UTR of coding transcripts were selected. There are 6793 common m6A 3'UTR peaks between WT26 and *Mettl14* KO and 2240 common m6A

3'UTR peaks between RBC and *Mettl3* KO data sets. The intensity of these intersected m6A peaks within a -3kb to +3kb region from the peak center is shown in the plots. The data represent the mean from n=2 biological replicates.

Supplementary Figure S8. Immunofluorescence and ChIP analysis for H3K9me3 in WT26 and *Mettl14* KO and in RBC and *Mettl3* KO ES cells. (A) IF images of WT26 and *Mettl14* KO and of RBC and *Mettl3* KO ES cells stained with DAPI and with α-H3K9me3 and α-HP1α antibodies. Scale bar = 5 µM. **(B)** Directed ChIP analysis for H3K9me3 accumulation at the MSR or LINE L1MdA 5'UTR repeats in WT26 and *Mettl14* KO and in RBC and *Mettl3* KO ES cells (top panels). As controls, H3K9me3 ChIP was also done for *Zfp180* exon 5 (positive control) and the *β-actin* gene promoter (negative control) (lower panels). Enrichment is calculated as the percentage of H3K9me3-enriched qPCR signal compared to input qPCR signal. The data represent the mean ± SD from n=2 biological replicates.

Supplementary Figure S9. Quantification of the subcellular distribution of *Gapdh, Hprt*, LINE L1MdA 5'UTR and MSR transcripts in WT26/RBC and in *Mettl14* /*Mettl3* KO ES cells. (A) Histogram showing the distribution of *Gapdh*, *Hprt*, LINE L1MdA 5'UTR and MSR transcripts in cytoplasmic (white), nucleoplasmic (grey) and chromatin (black) fractions of WT26, *Mettl14* KO, RBC and *Mettl3* KO ES cells. RNA was isolated from each of the subcellular fractions and specific transcripts were quantified by directed RT-qPCR. The relative abundance of transcripts in each subcellular fraction is displayed as the percentage of total transcripts in all three subcellular fractions. Statistical significance for differences in transcript distribution between cytoplasmic, nucleoplasmic and chromatin fractions within each of the *Gapdh*, *Hprt*, L1MdA 5'UTR and MSR data sets was confirmed by unpaired two-tailed t-test. For clarity, these p-values are not shown. Differences in transcript distribution between WT and mutant samples are statistically significant only for the increase in nucleoplasmic MSR transcripts in both *Mettl14* KO and *Mettl3* KO samples, which is highlighted in (B). **(B)** Zoom-in histogram of the relative abundance of *Gapdh, Hprt*, L1MdA 5'UTR and MSR transcripts in the nucleoplasmic fraction of WT26, *Mettl14* KO, RBC and *Mettl3* KO ES cells, extracted from the graph in (A). Statistical significance was analyzed by unpaired two-tailed t-test and the p-values are indicated. All data represent the mean from n=3 biological replicates.

Supplementary Figure S10. Characterization of the recombinant hybrid binding domain (HBD) of mouse RNase H1 as a new reagent for the detection of RNA:DNA hybrids. (A) Schematic diagram of the 6xHis-MBP-NLS-HBD(H1)-eGFP fusion construct that contains the hybrid binding domain (amino acids 27 to 76) of the mouse RNase H1 enzyme. The 6xHis-MBP-tag is on the N-terminus and the eGFP tag on the C-terminus. The dot indicates the AcTEV cleavage site and NLS is a nuclear localization signal. Also shown is the 6xHis-MBP-NLS-eGFP control construct. **(B)** Coomassie staining of purified HBD(H1)-eGFP (left) or eGFP (right) after AcTEV cleavage. Bands corresponding to the size of HBD(H1)-eGFP and eGFP are indicated with arrows. kDa = kilodalton. **(C)** RDIP (with HBD(H1)-eGFP) of chromatin-associated, phenol/chloroform-extracted NA from *p53*$^{-/-}$ control and *p53*$^{-/-}$*; Rnaseh2*$^{-/-}$ MEFs. Prior to the enrichment with HBD(H1)-eGFP, NA were either untreated or treated for for 2 h at 4°C with RNase H (Roche). After the enrichment with HBD(H1)-eGFP, RNA:DNA hybrids were detected

by RNA amplification using RT-qPCR. The data represent the mean ± SD from n=2 biological replicates. Statistical significance was determined by paired two-tailed t-test and the p-values are indicated.

Supplementary Figure S11. RDIP signal detection of MSR RNA:DNA hybrids by DNA vs. RNA amplification. (A) Workflow for the RDIP analysis to detect RNA:DNA MSR hybrids by DNA amplification. After enrichment with HBD(H1)-eGFP or with the S9.6 antibody, DNA was isolated and directly used for qPCR. The histogram shows the MSR RDIP signal for chromatin-associated, phenol/chloroform-extracted NA from WT26 ES cells. RDIP was performed with untreated or RNase H (Roche) treated (2 hours at 4°C) NA that were probed with HBD(H1)-eGFP (left panel) or with the S9.6 antibody (right panel). **(B)** Workflow for the RDIP analysis to detect RNA:DNA MSR hybrids by RNA amplification. After enrichment with HBD(H1)-eGFP or with the S9.6 antibody, RNA was isolated, double DNase digested and used for RT-qPCR. The histogram shows the MSR RDIP signal for chromatin-associated, phenol/chloroform-extracted NA from WT26 ES cells. The data are calculated as percentage of HBD(H1)-eGFP or S9.6-enriched signal compared to the input signal. The data represent the mean ± SD from n=3 biological replicates. Statistical significance was determined by paired two-tailed t-test and the p-values are indicated.

Supplementary Figure S12. Detection of RNA:DNA hybrids by RDIP with S9.6 antibody.
(A) Electrophoretic mobility shift assay (EMSA) with S9.6 antibody and RNA:DNA hybrids that were generated by annealing ssRNA (35 nt) or ssDNA oligonucleotides (35 nt) from MSR subrepeat 2. RNA:DNA hybrids contain either unmodified (unmod) or 5mC- (5mC) or m6A-modified (m6A) RNA oligonucleotides. **(B)** Histogram showing RDIP (using the S9.6 antibody) of chromatin-associated, phenol/chloroform extracted NA from WT26, *Mettl14* KO, RBC and *Mettl3* KO ES cells. Prior to the enrichment with the S9.6 antibody, NA were either untreated (grey or hatched bars) or incubated for 2 hours at 4°C with RNase H (Roche) (white bars). MSR (left) and LINE L1MdA 5'UTR (right) RNA:DNA hybrids were detected by RNA amplification using RT-qPCR. The data are calculated as percentage of S9.6 enriched signal compared to the input signal in each sample. The data represent the mean ± SD from n=5 biological replicates. Statistical significance between untreated and RNase H treated samples was determined by paired two-tailed t-test and the p-values are indicated. Statistical significance between untreated wt and untreated mutant samples was determined by unpaired two tailed t-test and the p-values are indicated.

Supplementary Figure S13. *In vitro* RNA:DNA hybrid formation. (A) Diagram showing the sequence of a 35 nt RNA oligonucleotide derived from the subrepeat 2/3 of the MSR. This RNA oligonucleotide is in the forward orientiation (MSR-SR2/3-FOR) and contains 10 adenosines, which were either left unmodified or replaced by m6A (indicated by blue hexagon). **(B)** Constant (5 μM) concentration of complementary ssDNA oligonucleotides were annealed to increasing (0.1–10 μM) concentrations of MSR-SR2/3-FOR or MSR-SR2/3-FOR(m6A) RNA oligonucleotides, resolved on polyacrylamide gel and stained with SYBR Gold (upper panel). The nucleic acids were then blotted and RNA:DNA hybrids were detected with the S9.6 antibody (lower panel). **(C)** The same as in (B), but increasing (0.5–10 μM) concentrations of MSR-SR2/3-FOR or MSR-SR2/3-FOR(m6A) RNA oligonucleotides were annealed to

a constant concentration (5 μM) of complementary dsDNA, probing the ability of the RNA to invade a DNA duplex and to form RNA:DNA hybrids. We could not perform the same experiment with the HBD(H1)-eGFP reagent, as the recombinant HBD(H1)-eGFP did not work on Hybond N+ (GE Healthcare) membranes.

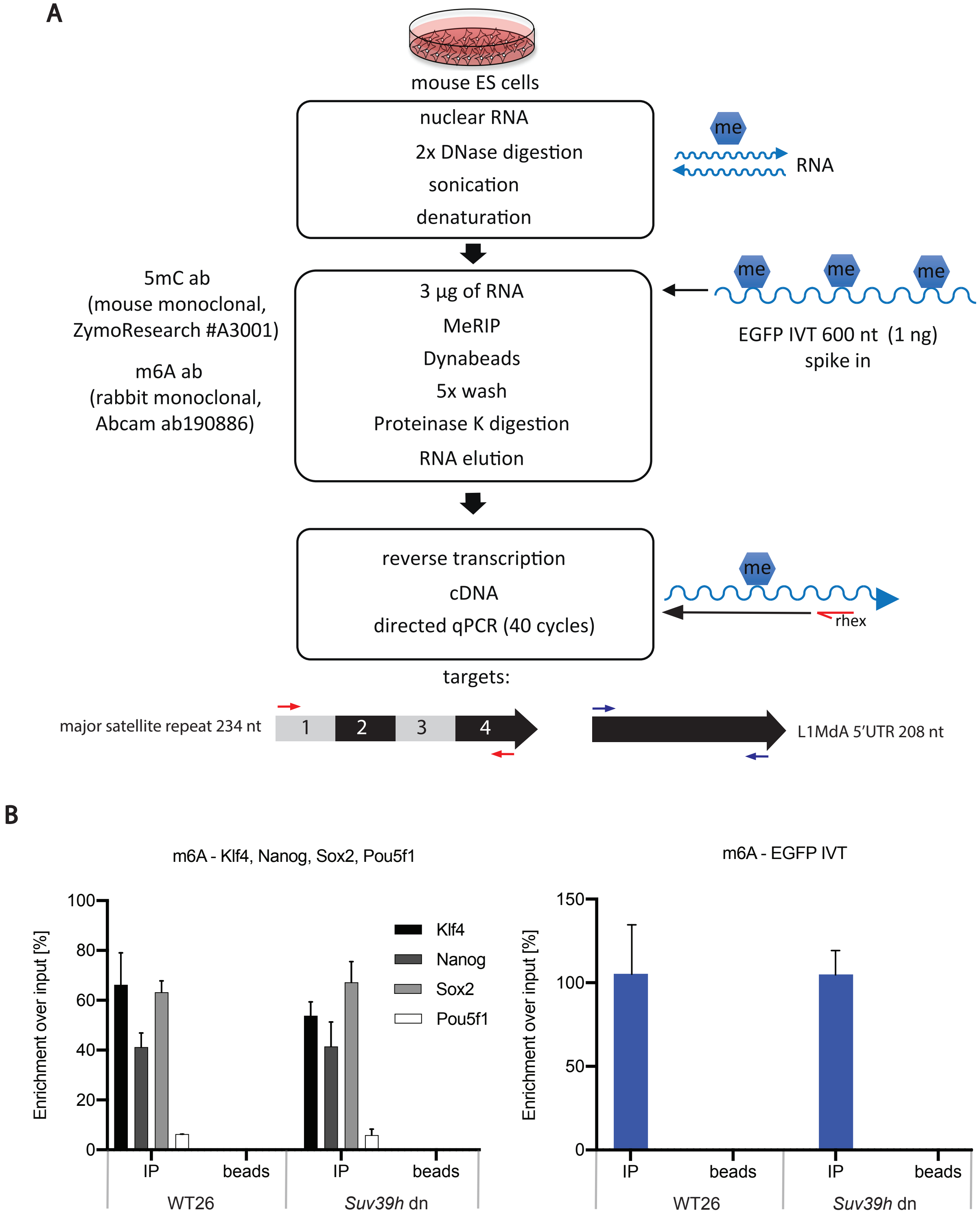
A
mouse ES cells
nuclear RNA
2x DNase digestion
sonication
denaturation
me
RNA
5mC ab
(mouse monoclonal,
ZymoResearch #A3001)
m6A ab
(rabbit monoclonal,
Abcam ab190886)
3 µg of RNA
MeRIP
Dynabeads
5x wash
Proteinase K digestion
RNA elution
me
me
me
EGFP IVT 600 nt (1 ng)
spike in
reverse transcription
cDNA
directed qPCR (40 cycles)
me
rhex
targets:
major satellite repeat 234 nt
1
2
3
4
L1MdA 5'UTR 208 nt
B
m6A - Klf4, Nanog, Sox2, Pou5f1
Enrichment over input [%]
100
80
60
40
20
0
Klf4
Nanog
Sox2
Pou5f1
IP
beads
IP
beads
WT26
Suv39h dn
m6A - EGFP IVT
Enrichment over input [%]
150
100
50
0
IP
beads
IP
beads
WT26
Suv39h dn

Figure S1

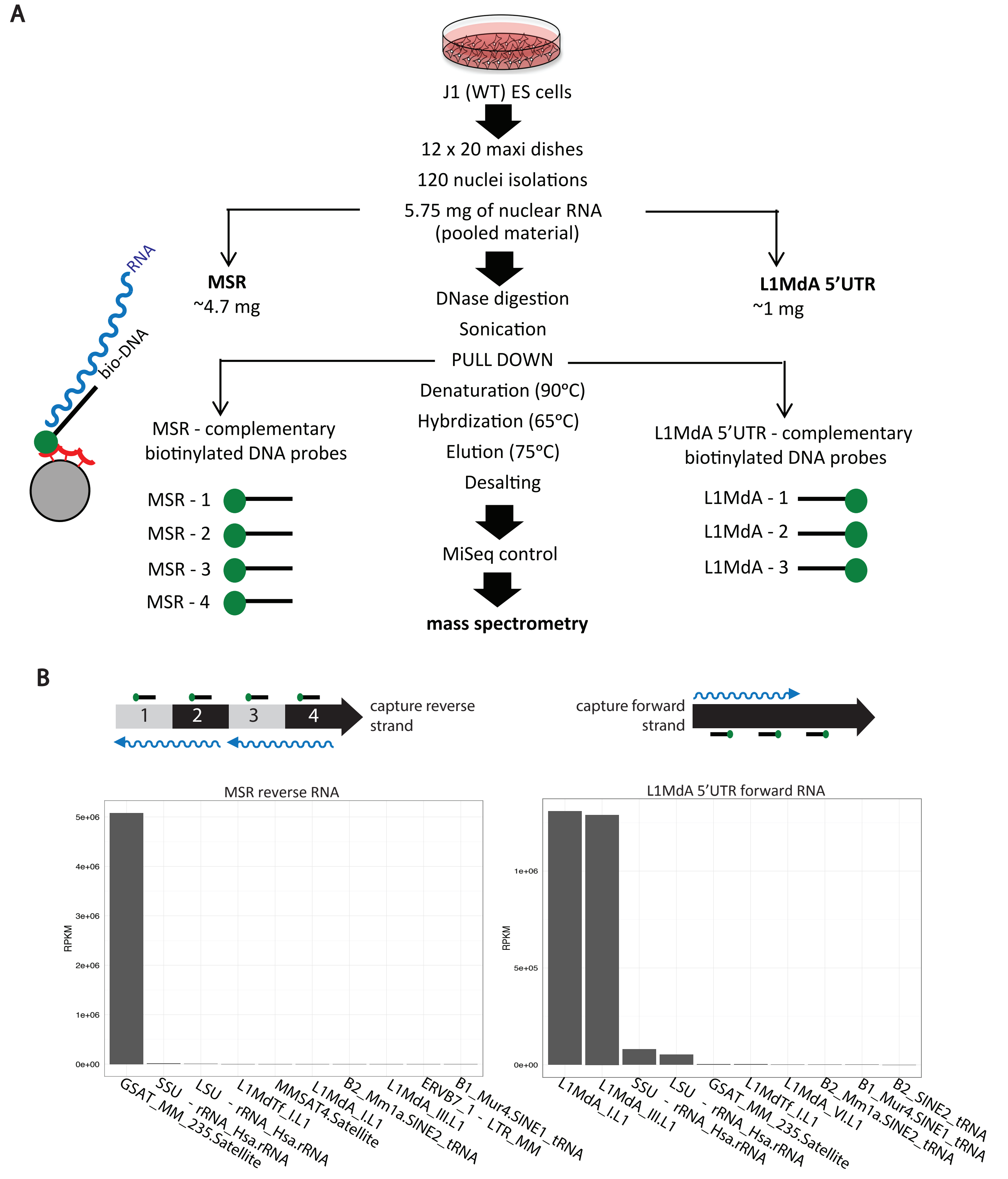
A
J1 (WT) ES cells
12 x 20 maxi dishes
120 nuclei isolations
5.75 mg of nuclear RNA
(pooled material)
MSR
~4.7 mg
L1MdA 5'UTR
~1 mg
DNase digestion
Sonication
PULL DOWN
Denaturation (90°C)
Hybrdization (65°C)
Elution (75°C)
Desalting
MiSeq control
mass spectrometry
RNA
bio-DNA
MSR - complementary
biotinylated DNA probes
MSR - 1
MSR - 2
MSR - 3
MSR - 4
L1MdA 5'UTR - complementary
biotinylated DNA probes
L1MdA - 1
L1MdA - 2
L1MdA - 3
B
capture reverse
strand
capture forward
strand
MSR reverse RNA
L1MdA 5'UTR forward RNA
RPKM
5e+06
4e+06
3e+06
2e+06
1e+06
0e+00
1e+06
5e+05
GSAT_MM_235.Satellite
SSU - rRNA_Hsa.rRNA
LSU - rRNA_Hsa.rRNA
L1MdTf_I.L1
MMSAT4.Satellite
L1MdA_I.L1
B2_Mm1a.SINE2_tRNA
L1MdA_III.L1
ERVB7_1 - LTR_MM
B1_Mur4.SINE1_tRNA
L1MdA_VI.L1
B2_SINE2_tRNA

Figure S2

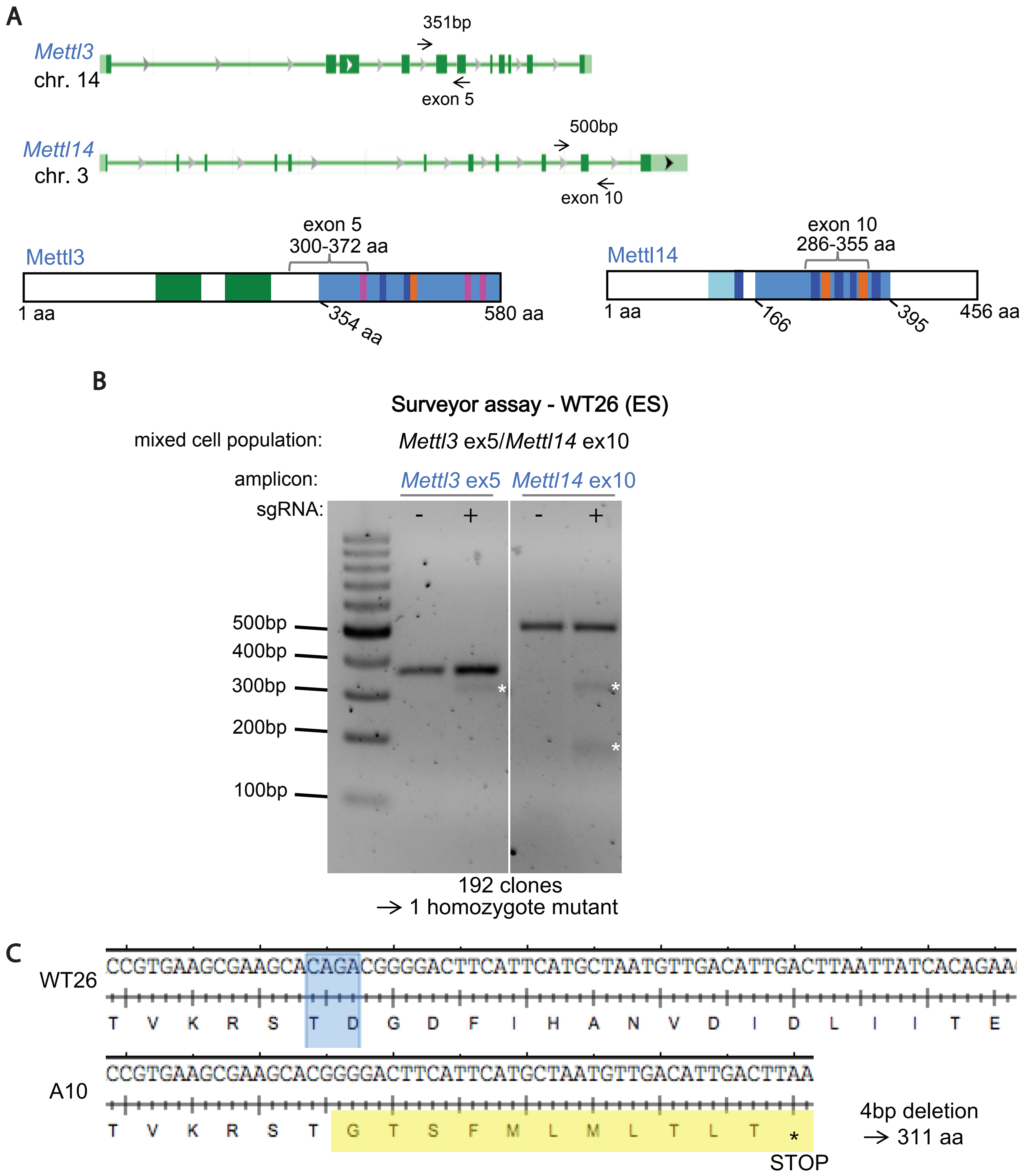
A
Mettl3
chr. 14
351bp
exon 5
Mettl14
chr. 3
500bp
exon 10
Mettl3
exon 5
300-372 aa
1 aa
354 aa
580 aa
Mettl14
exon 10
286-355 aa
1 aa
166
395
456 aa
B
Surveyor assay - WT26 (ES)
mixed cell population:
Mettl3 ex5/Mettl14 ex10
amplicon:
Mettl3 ex5
Mettl14 ex10
sgRNA:
- + - +
500bp
400bp
300bp
200bp
100bp
192 clones
→ 1 homozygote mutant
C
WT26
CCGTGAAGCGAAGCACAGACGGGGACTTCATTCATGCTAATGTTGACATTGACTTAATTATCACAGAA
T V K R S T D G D F I H A N V D I D L I I T E
A10
CCGTGAAGCGAAGCACGGGGACTTCATTCATGCTAATGTTGACATTGACTTAA
T V K R S T G T S F M L M L T L T *
STOP
4bp deletion
→ 311 aa

Figure S3

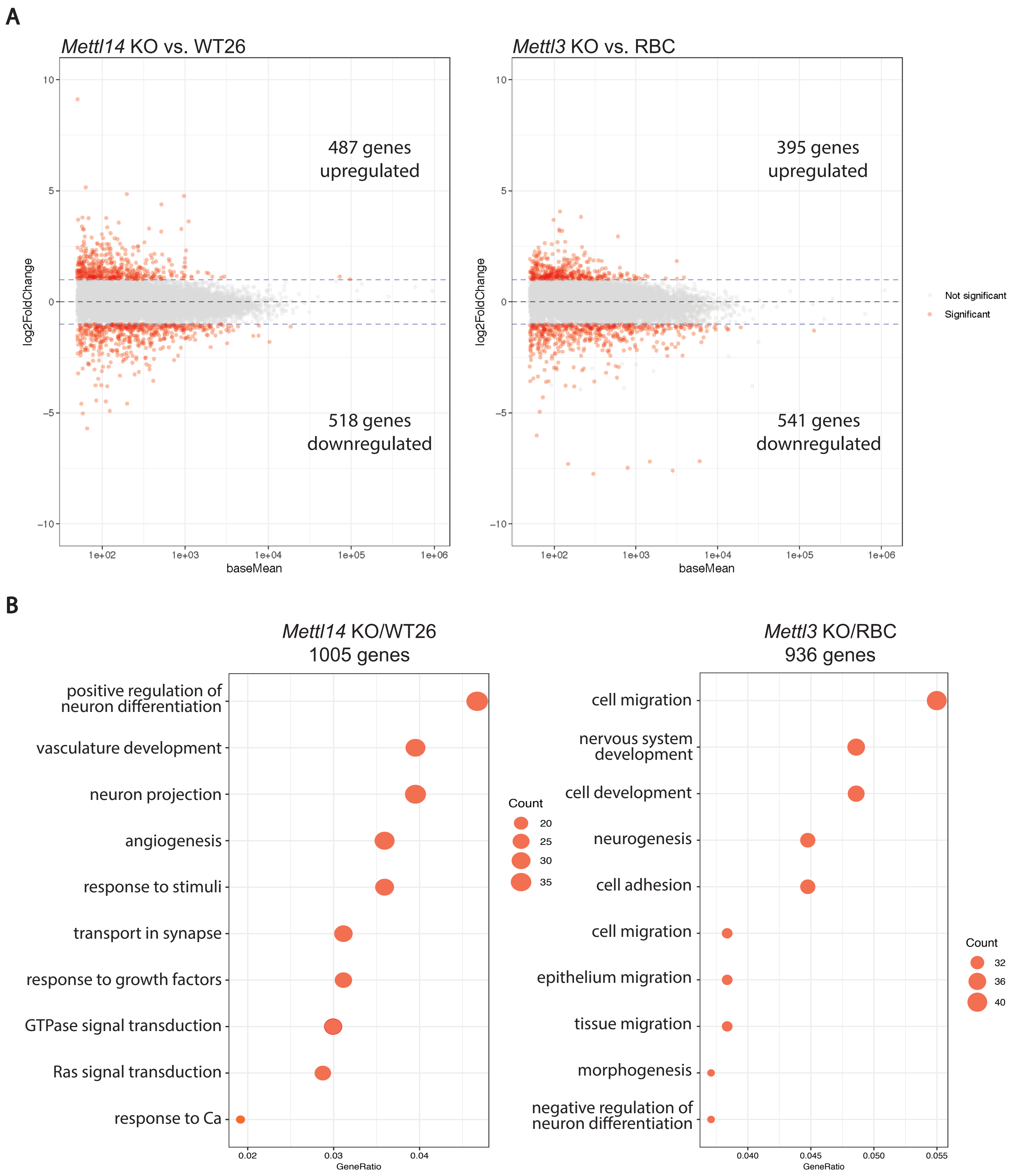

A
Mettl14 KO vs. WT26
487 genes upregulated
518 genes downregulated
log2FoldChange
baseMean
Mettl3 KO vs. RBC
395 genes upregulated
541 genes downregulated
Not significant
Significant
B
Mettl14 KO/WT26
1005 genes
positive regulation of neuron differentiation
vasculature development
neuron projection
angiogenesis
response to stimuli
transport in synapse
response to growth factors
GTPase signal transduction
Ras signal transduction
response to Ca
GeneRatio
Count
Mettl3 KO/RBC
936 genes
cell migration
nervous system development
cell development
neurogenesis
cell adhesion
cell migration
epithelium migration
tissue migration
morphogenesis
negative regulation of neuron differentiation
GeneRatio
Count

Figure S4

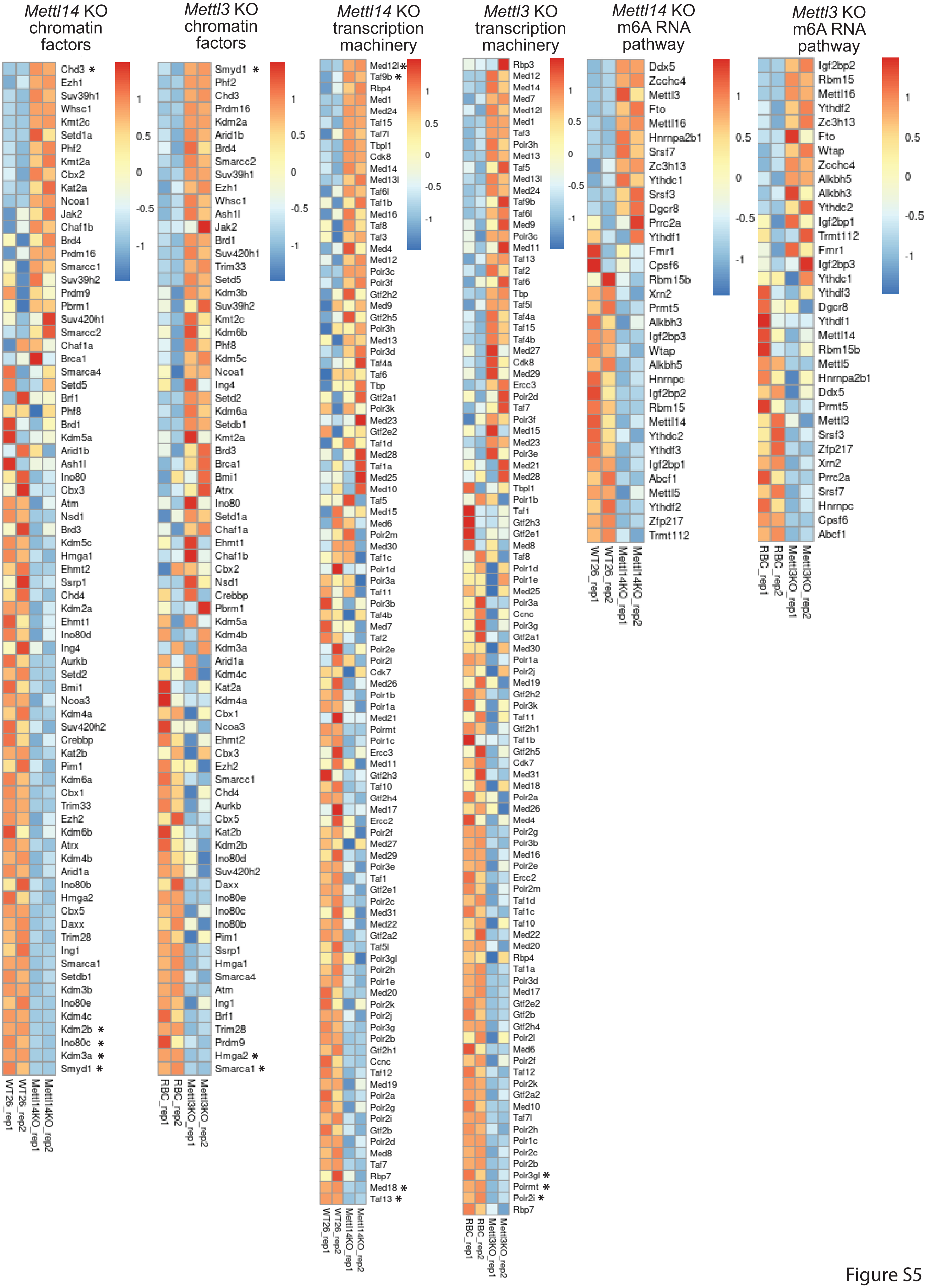
Mettl14 KO chromatin factors
1
0.5
0
-0.5
-1
Chd3 *
Ezh1
Suv39h1
Whsc1
Kmt2c
Setd1a
Phf2
Kmt2a
Cbx2
Kat2a
Ncoa1
Jak2
Chaf1b
Brd4
Prdm16
Smarcc1
Suv39h2
Prdm9
Pbrm1
Suv420h1
Smarcc2
Chaf1a
Brca1
Smarca4
Setd5
Brf1
Phf8
Brd1
Kdm5a
Arid1b
Ash1l
Ino80
Cbx3
Atm
Nsd1
Brd3
Kdm5c
Hmga1
Ehmt2
Ssrp1
Chd4
Kdm2a
Ehmt1
Ino80d
Ing4
Aurkb
Setd2
Bmi1
Ncoa3
Kdm4a
Suv420h2
Crebbp
Kat2b
Pim1
Kdm6a
Cbx1
Trim33
Ezh2
Kdm6b
Atrx
Kdm4b
Arid1a
Ino80b
Hmga2
Cbx5
Daxx
Trim28
Ing1
Smarca1
Setdb1
Kdm3b
Ino80e
Kdm4c
Kdm2b *
Ino80c *
Kdm3a *
Smyd1 *
WT26_rep1
WT26_rep2
Mettl14KO_rep1
Mettl14KO_rep2
Mettl3 KO chromatin factors
1
0.5
0
-0.5
-1
Smyd1 *
Phf2
Chd3
Prdm16
Kdm2a
Arid1b
Brd4
Smarcc2
Suv39h1
Ezh1
Whsc1
Ash1l
Jak2
Brd1
Suv420h1
Trim33
Setd5
Kdm3b
Suv39h2
Kmt2c
Kdm6b
Phf8
Kdm5c
Ncoa1
Ing4
Setd2
Kdm6a
Setdb1
Kmt2a
Brd3
Brca1
Bmi1
Atrx
Ino80
Setd1a
Chaf1a
Ehmt1
Chaf1b
Cbx2
Nsd1
Crebbp
Pbrm1
Kdm5a
Kdm4b
Kdm3a
Arid1a
Kdm4c
Kat2a
Kdm4a
Cbx1
Ncoa3
Ehmt2
Cbx3
Ezh2
Smarcc1
Chd4
Aurkb
Cbx5
Kat2b
Kdm2b
Ino80d
Suv420h2
Daxx
Ino80e
Ino80c
Ino80b
Pim1
Ssrp1
Hmga1
Smarca4
Atm
Ing1
Brf1
Trim28
Prdm9
Hmga2 *
Smarca1 *
RBC_rep1
RBC_rep2
Mettl3KO_rep1
Mettl3KO_rep2
Mettl14 KO transcription machinery
1
0.5
0
-0.5
-1
Med12l *
Taf9b *
Rbp4
Med1
Med24
Taf15
Taf7l
Tbpl1
Cdk8
Med14
Med13l
Taf6l
Taf1b
Med16
Taf8
Taf3
Med4
Med12
Polr3c
Polr3f
Gtf2h2
Med9
Gtf2h5
Polr3h
Med13
Polr3d
Taf4a
Taf6
Tbp
Gtf2a1
Polr3k
Med23
Gtf2e2
Taf1d
Med28
Taf1a
Med25
Med10
Taf5
Med15
Med6
Polr2m
Med30
Taf1c
Polr1d
Polr3a
Taf11
Polr3b
Taf4b
Med7
Taf2
Polr2e
Polr2l
Cdk7
Med26
Polr1b
Polr1a
Med21
Polrmt
Polr1c
Ercc3
Med11
Gtf2h3
Taf10
Gtf2h4
Med17
Ercc2
Polr2f
Med27
Med29
Polr3e
Taf1
Gtf2e1
Polr2c
Med31
Med22
Gtf2a2
Taf5l
Polr3gl
Polr2h
Polr1e
Med20
Polr2k
Polr2j
Polr3g
Polr2b
Gtf2h1
Ccnc
Taf12
Med19
Polr2a
Polr2g
Polr2i
Gtf2b
Polr2d
Med8
Taf7
Rbp7
Med18 *
Taf13 *
WT26_rep1
WT26_rep2
Mettl14KO_rep1
Mettl14KO_rep2
Mettl3 KO transcription machinery
1
0.5
0
-0.5
-1
Rbp3
Med12
Med14
Med7
Med12l
Med1
Taf3
Polr3h
Med13
Taf5
Med13l
Med24
Taf9b
Taf6l
Med9
Polr3c
Med11
Taf13
Taf2
Taf6
Tbp
Taf5l
Taf4a
Taf15
Taf4b
Med27
Cdk8
Med29
Ercc3
Polr2d
Taf7
Polr3f
Med15
Med23
Polr3e
Med21
Med28
Tbpl1
Polr1b
Taf1
Gtf2h3
Gtf2e1
Med8
Taf8
Polr1d
Polr1e
Med25
Polr3a
Ccnc
Polr3g
Gtf2a1
Med30
Polr1a
Polr2j
Med19
Gtf2h2
Polr3k
Taf11
Gtf2h1
Taf1b
Gtf2h5
Cdk7
Med31
Med18
Polr2a
Med26
Med4
Polr2g
Polr3b
Med16
Polr2e
Ercc2
Polr2m
Taf1d
Taf1c
Taf10
Med22
Med20
Rbp4
Taf1a
Polr3d
Med17
Gtf2e2
Gtf2b
Gtf2h4
Polr2l
Med6
Polr2f
Taf12
Polr2k
Gtf2a2
Med10
Taf7l
Polr2h
Polr1c
Polr2c
Polr2b
Polr3gl *
Polrmt *
Polr2i *
Rbp7
RBC_rep1
RBC_rep2
Mettl3KO_rep1
Mettl3KO_rep2
Mettl14 KO m6A RNA pathway
1
0.5
0
-0.5
-1
Ddx5
Zcchc4
Mettl3
Fto
Mettl16
Hnrnpa2b1
Srsf7
Zc3h13
Ythdc1
Srsf3
Dgcr8
Prrc2a
Ythdf1
Fmr1
Cpsf6
Rbm15b
Xrn2
Prmt5
Alkbh3
Igf2bp3
Wtap
Alkbh5
Hnrnpc
Igf2bp2
Rbm15
Mettl14
Ythdc2
Ythdf3
Igf2bp1
Abcf1
Mettl5
Ythdf2
Zfp217
Trmt112
WT26_rep1
WT26_rep2
Mettl14KO_rep1
Mettl14KO_rep2
Mettl3 KO m6A RNA pathway
1
0.5
0
-0.5
-1
Igf2bp2
Rbm15
Mettl16
Ythdf2
Zc3h13
Fto
Wtap
Zcchc4
Alkbh5
Alkbh3
Ythdc2
Igf2bp1
Trmt112
Fmr1
Igf2bp3
Ythdc1
Ythdf3
Dgcr8
Ythdf1
Mettl14
Rbm15b
Mettl5
Hnrnpa2b1
Ddx5
Prmt5
Mettl3
Srsf3
Zfp217
Xrn2
Prrc2a
Srsf7
Hnrnpc
Cpsf6
Abcf1
RBC_rep1
RBC_rep2
Mettl3KO_rep1
Mettl3KO_rep2

Figure S5

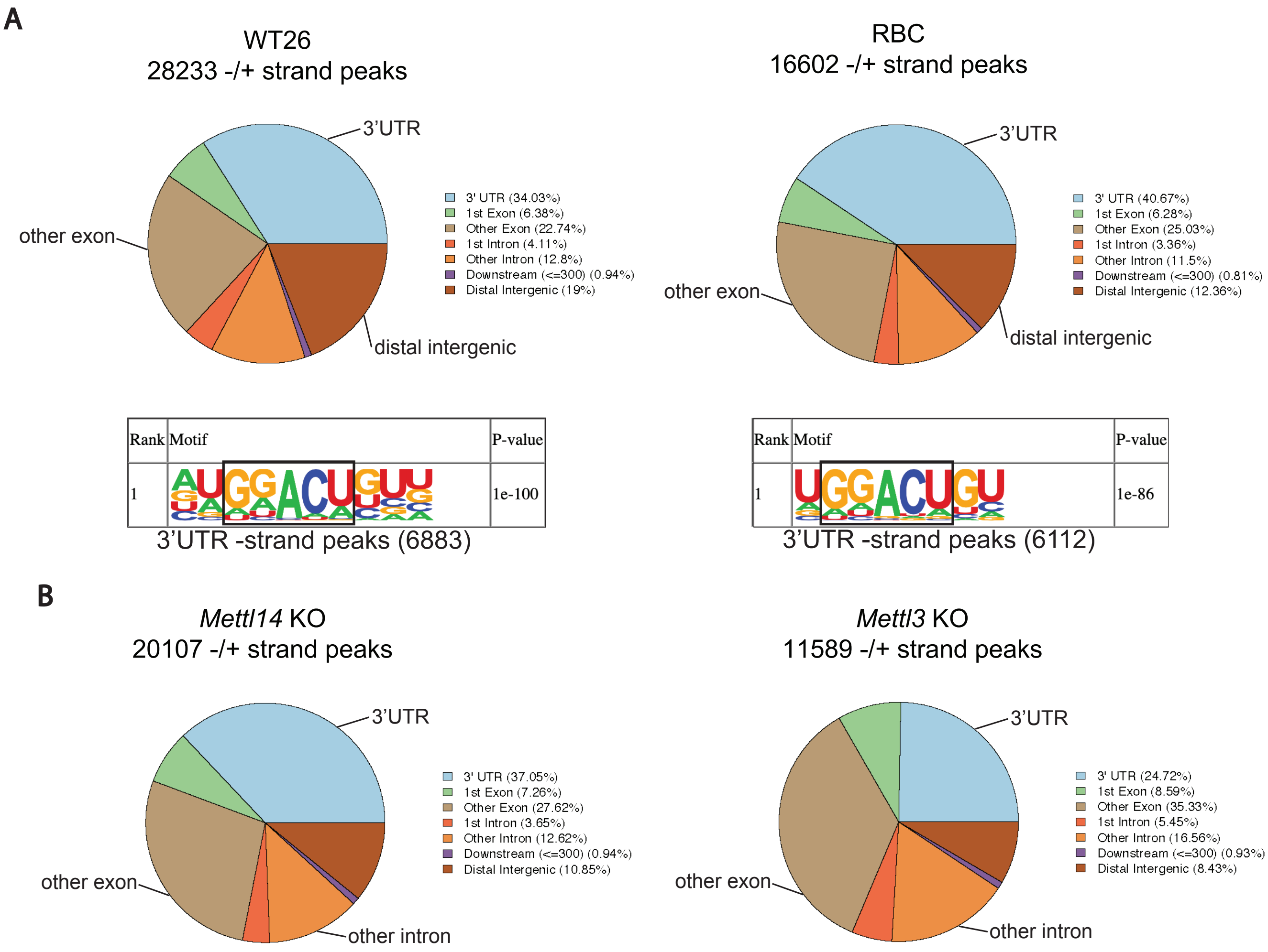
A
WT26
28233 -/+ strand peaks
3'UTR
other exon
distal intergenic
3' UTR (34.03%)
1st Exon (6.38%)
Other Exon (22.74%)
1st Intron (4.11%)
Other Intron (12.8%)
Downstream (<=300) (0.94%)
Distal Intergenic (19%)
Rank
Motif
P-value
1
1e-100
3'UTR -strand peaks (6883)
RBC
16602 -/+ strand peaks
3'UTR
other exon
distal intergenic
3' UTR (40.67%)
1st Exon (6.28%)
Other Exon (25.03%)
1st Intron (3.36%)
Other Intron (11.5%)
Downstream (<=300) (0.81%)
Distal Intergenic (12.36%)
Rank
Motif
P-value
1
1e-86
3'UTR -strand peaks (6112)
B
Mettl14 KO
20107 -/+ strand peaks
3'UTR
other exon
other intron
3' UTR (37.05%)
1st Exon (7.26%)
Other Exon (27.62%)
1st Intron (3.65%)
Other Intron (12.62%)
Downstream (<=300) (0.94%)
Distal Intergenic (10.85%)
Mettl3 KO
11589 -/+ strand peaks
3'UTR
other exon
other intron
3' UTR (24.72%)
1st Exon (8.59%)
Other Exon (35.33%)
1st Intron (5.45%)
Other Intron (16.56%)
Downstream (<=300) (0.93%)
Distal Intergenic (8.43%)

Figure S6

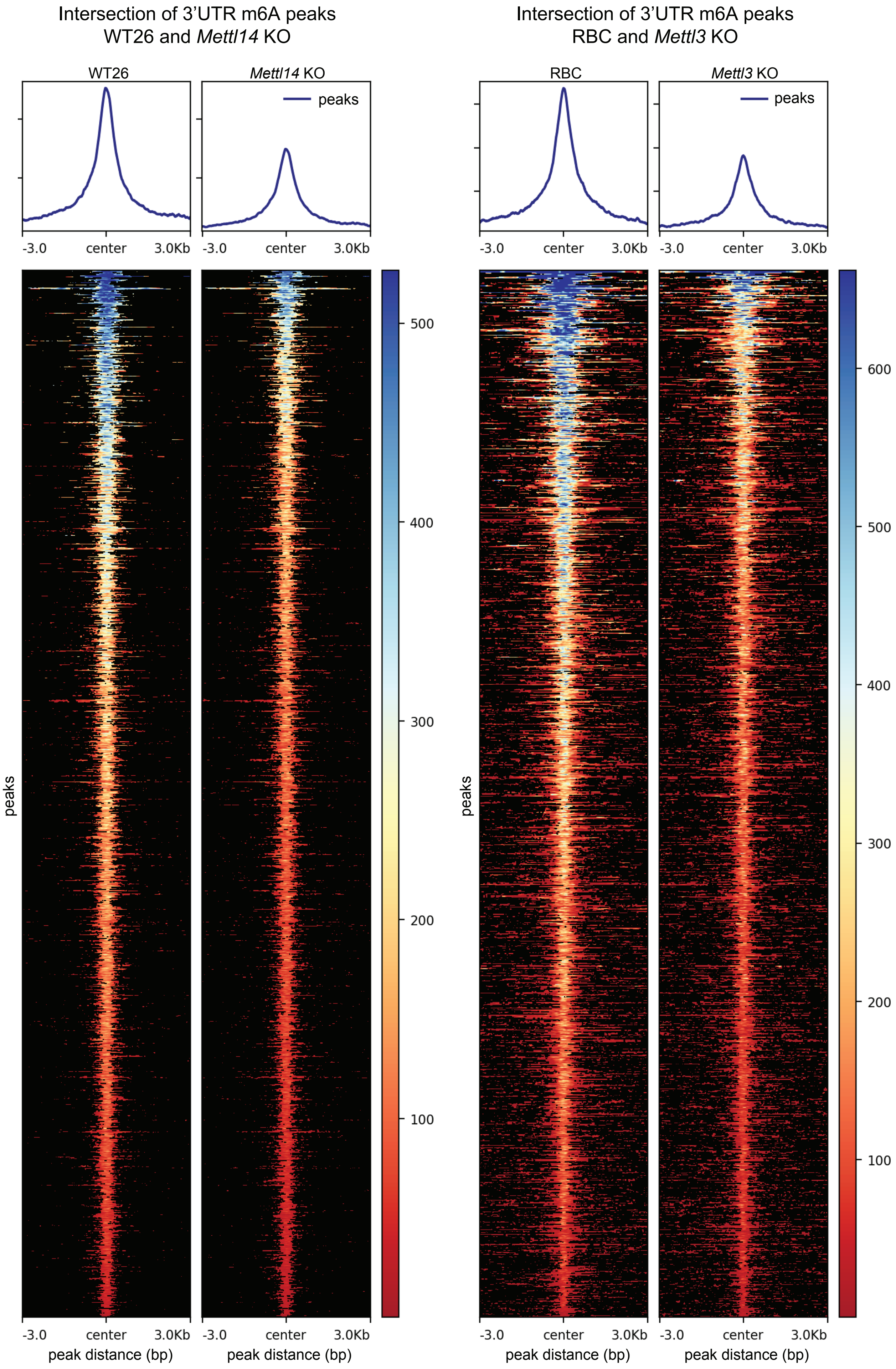

Intersection of 3'UTR m6A peaks
WT26 and *Mettl14* KO
WT26
Mettl14 KO
peaks
-3.0
center
3.0Kb
500
400
300
200
100
peak distance (bp)
Intersection of 3'UTR m6A peaks
RBC and *Mettl3* KO
RBC
Mettl3 KO
600

Figure S7

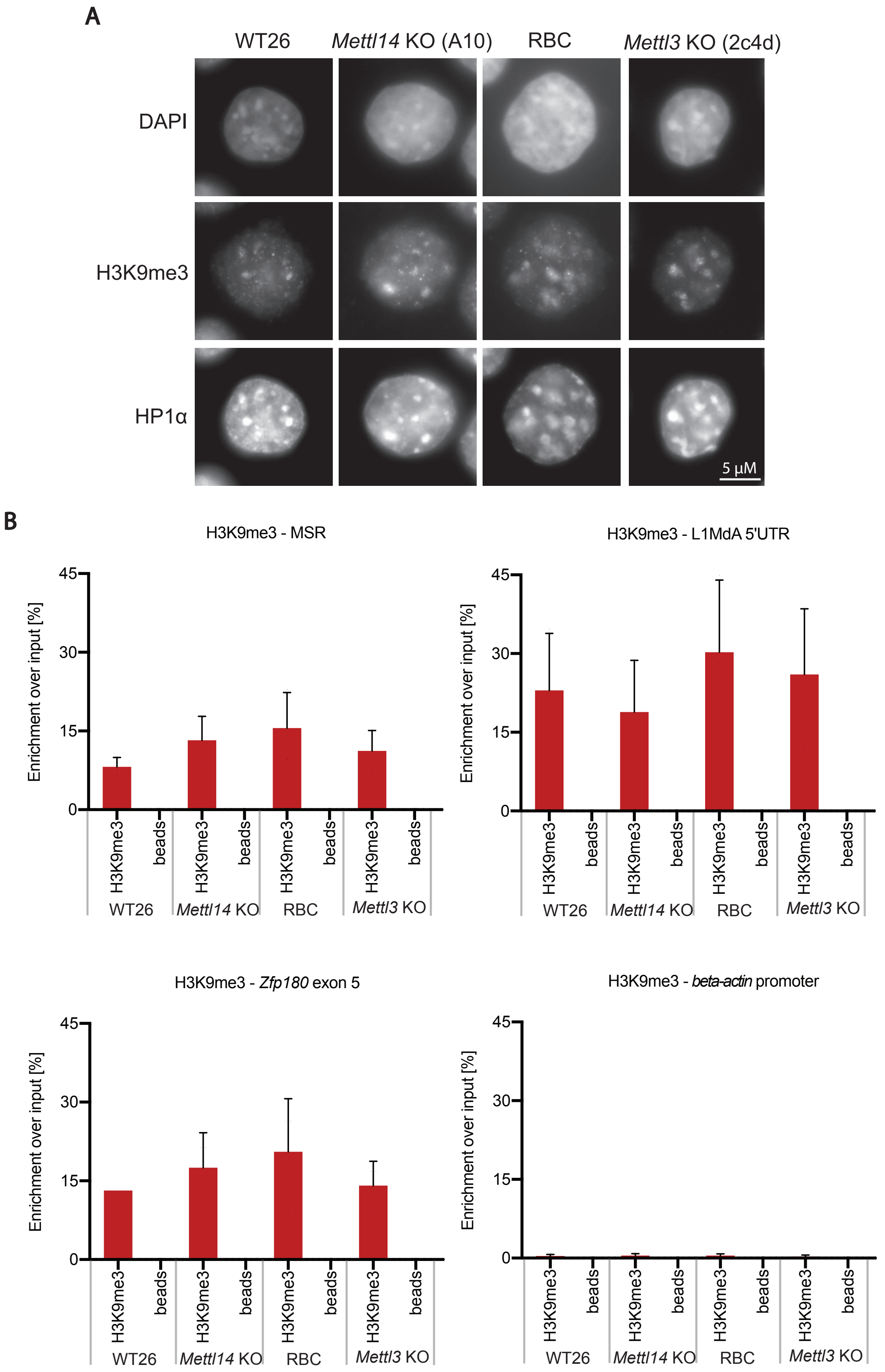
A
WT26
Mettl14 KO (A10)
RBC
Mettl3 KO (2c4d)
DAPI
H3K9me3
HP1α
5 μM
B
H3K9me3 - MSR
H3K9me3 - L1MdA 5'UTR
H3K9me3 - Zfp180 exon 5
H3K9me3 - beta-actin promoter
Enrichment over input [%]
45
30
15
0
H3K9me3
beads
WT26
Mettl14 KO
RBC
Mettl3 KO

Figure S8

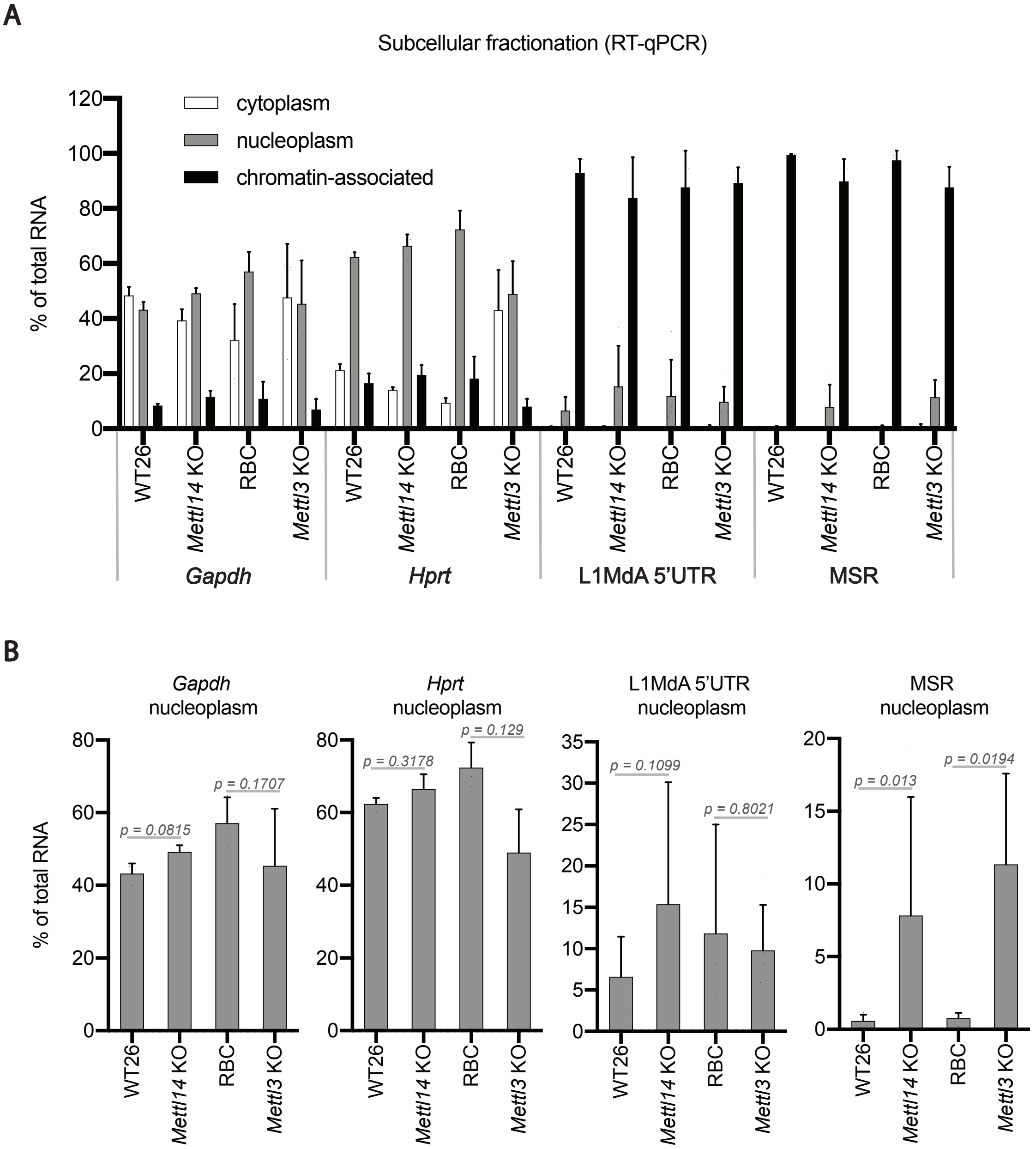
A
Subcellular fractionation (RT-qPCR)
cytoplasm
nucleoplasm
chromatin-associated
% of total RNA
WT26
Mettl14 KO
RBC
Mettl3 KO
Gapdh
Hprt
L1MdA 5'UTR
MSR
B
Gapdh nucleoplasm
Hprt nucleoplasm
L1MdA 5'UTR nucleoplasm
MSR nucleoplasm
p = 0.0815
p = 0.1707
p = 0.3178
p = 0.129
p = 0.1099
p = 0.8021
p = 0.013
p = 0.0194

Figure S9

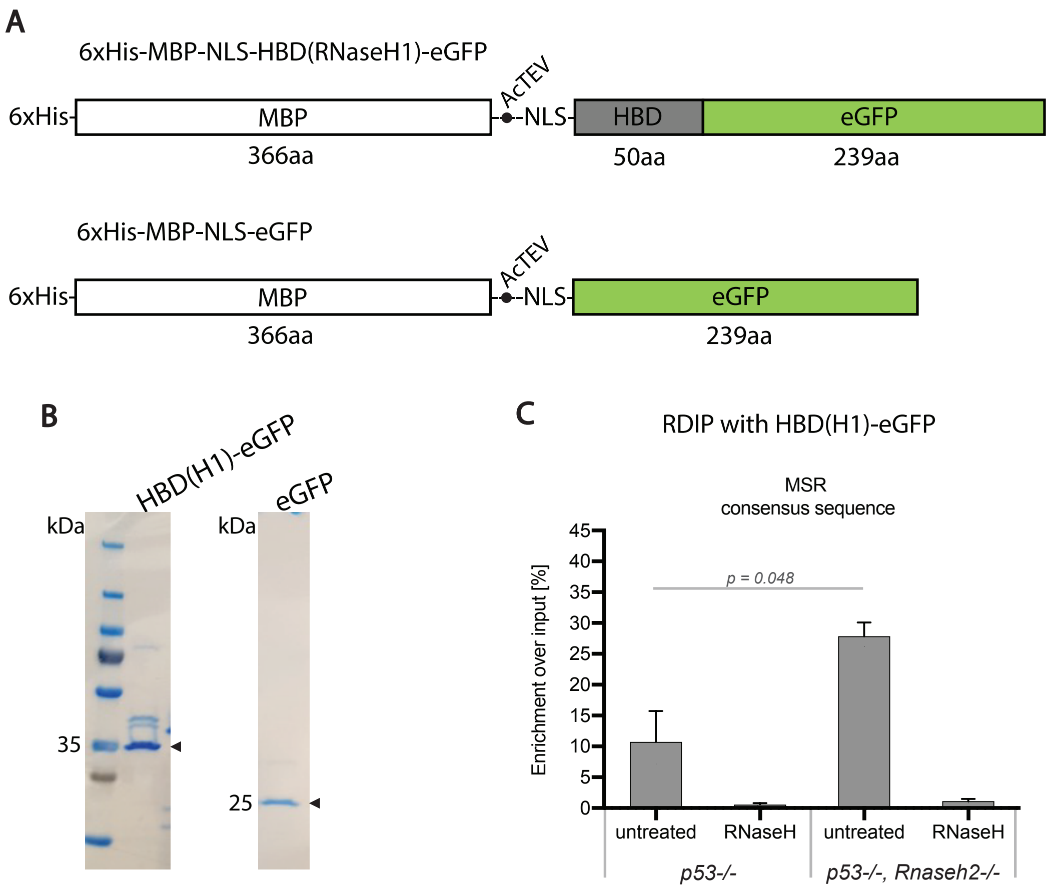
A
6xHis-MBP-NLS-HBD(RNaseH1)-eGFP
6xHis
MBP
AcTEV
NLS
HBD
eGFP
366aa
50aa
239aa
6xHis-MBP-NLS-eGFP
6xHis
MBP
AcTEV
NLS
eGFP
366aa
239aa
B
HBD(H1)-eGFP
eGFP
kDa
35
kDa
25
C
RDIP with HBD(H1)-eGFP
MSR
consensus sequence
Enrichment over input [%]
45
40
35
30
25
20
15
10
5
0
p = 0.048
untreated
RNaseH
untreated
RNaseH
p53-/-
p53-/-, Rnaseh2-/-

Figure S10

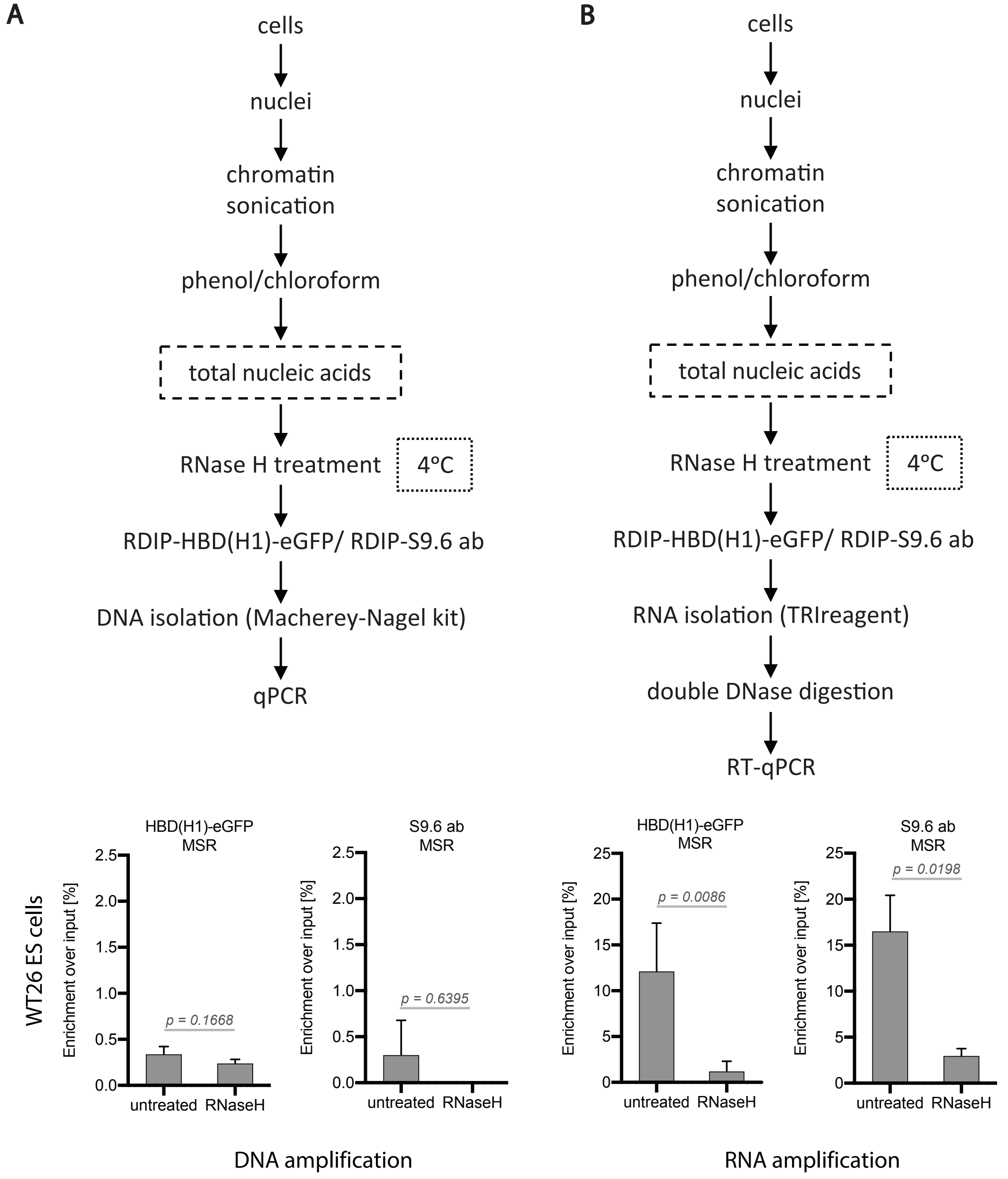
A
cells
nuclei
chromatin sonication
phenol/chloroform
total nucleic acids
RNase H treatment
4°C
RDIP-HBD(H1)-eGFP/ RDIP-S9.6 ab
DNA isolation (Macherey-Nagel kit)
qPCR
B
cells
nuclei
chromatin sonication
phenol/chloroform
total nucleic acids
RNase H treatment
4°C
RDIP-HBD(H1)-eGFP/ RDIP-S9.6 ab
RNA isolation (TRIreagent)
double DNase digestion
RT-qPCR
WT26 ES cells
HBD(H1)-eGFP MSR
S9.6 ab MSR
Enrichment over input [%]
p = 0.1668
p = 0.6395
p = 0.0086
p = 0.0198
untreated
RNaseH
DNA amplification
RNA amplification

Figure S11

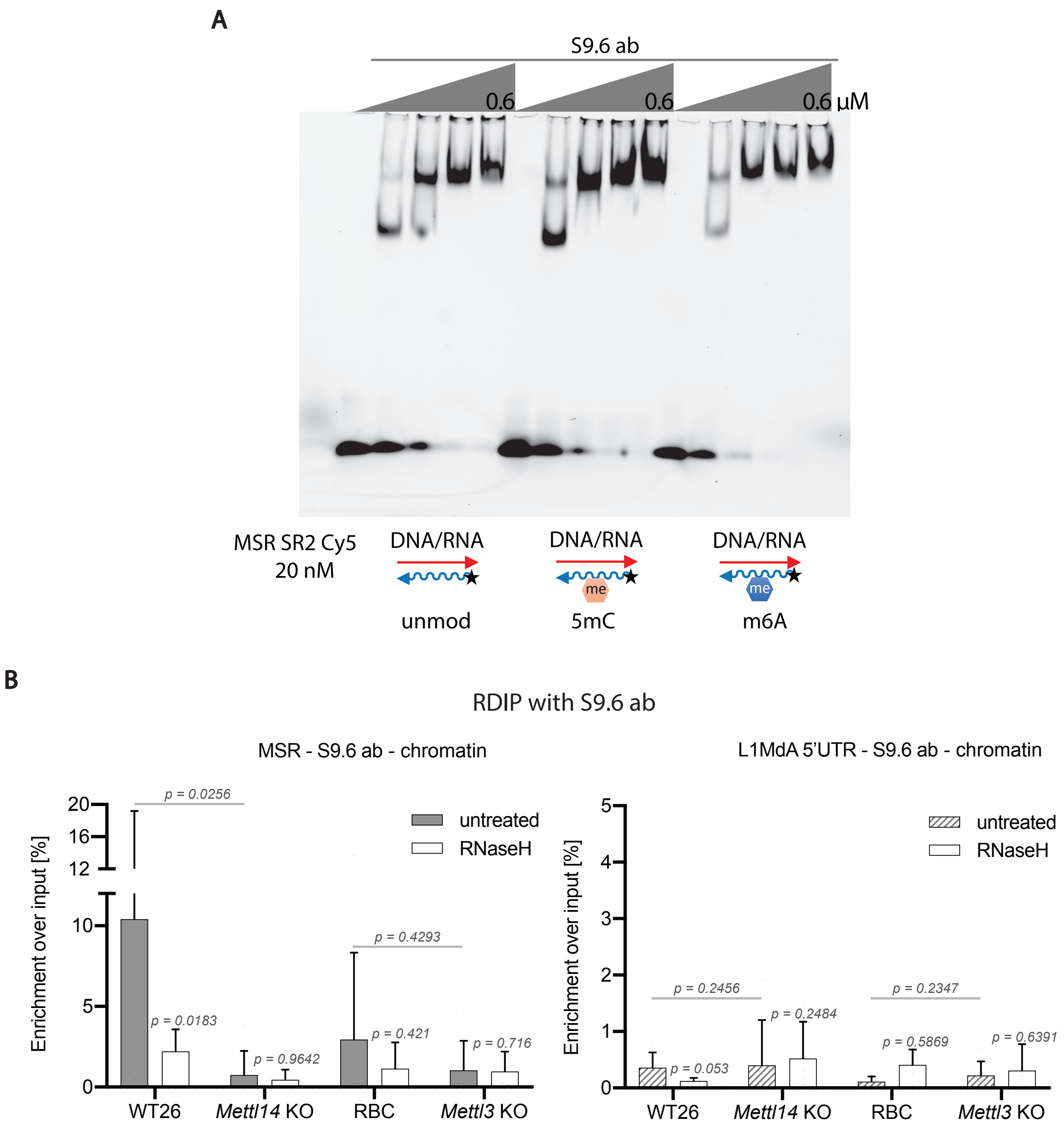
A
S9.6 ab
0.6
0.6
0.6 µM
MSR SR2 Cy5
20 nM
DNA/RNA
DNA/RNA
DNA/RNA
me
me
unmod
5mC
m6A
B
RDIP with S9.6 ab
MSR - S9.6 ab - chromatin
L1MdA 5'UTR - S9.6 ab - chromatin
Enrichment over input [%]
untreated
RNaseH
p = 0.0256
p = 0.0183
p = 0.9642
p = 0.4293
p = 0.421
p = 0.716
p = 0.2456
p = 0.053
p = 0.2484
p = 0.2347
p = 0.5869
p = 0.6391
WT26
Mettl14 KO
RBC
Mettl3 KO

Figure S12

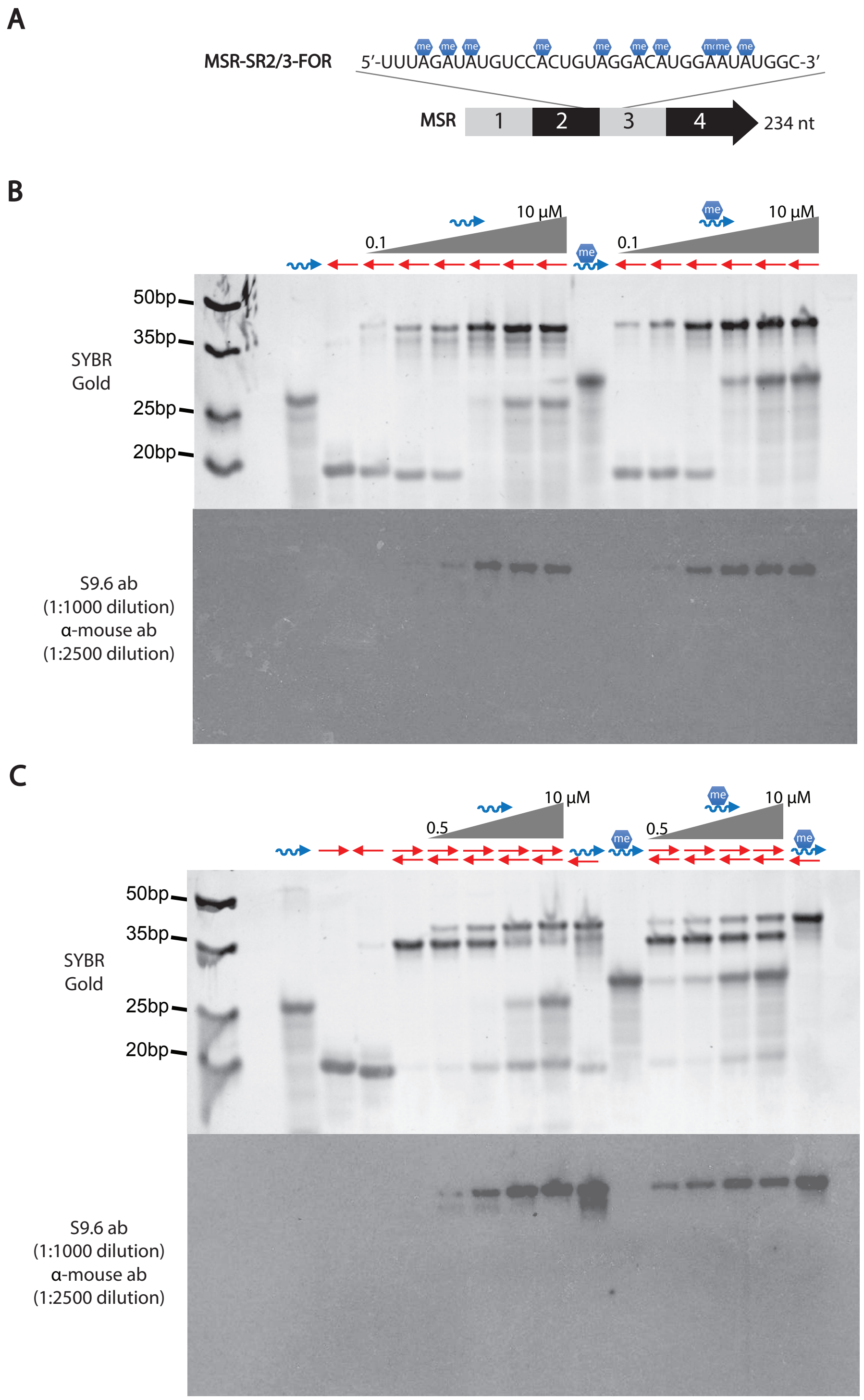
A
MSR-SR2/3-FOR
5′-UUUAGAUAUGUCCACUGUAGGACAUGGAAUAUGGC-3′
MSR
1
2
3
4
234 nt
B
10 µM
0.1
50bp
35bp
25bp
20bp
SYBR Gold
S9.6 ab (1:1000 dilution) α-mouse ab (1:2500 dilution)
C
10 µM
0.5
50bp
35bp
25bp
20bp
SYBR Gold
S9.6 ab (1:1000 dilution) α-mouse ab (1:2500 dilution)

Figure S13
